# *Enterobacter bugandensis*: a novel enterobacterial species associated with severe clinical infection

**DOI:** 10.1038/s41598-018-23069-z

**Published:** 2018-03-29

**Authors:** Niladri Bhusan Pati, Swapnil Prakash Doijad, Tilman Schultze, Gopala Krishna Mannala, Yancheng Yao, Sangeeta Jaiswal, Daniel Ryan, Mrutyunjay Suar, Konrad Gwozdzinski, Boyke Bunk, Mobarak Abu Mraheil, Mohamed A. Marahiel, Julian D. Hegemann, Cathrin Spröer, Alexander Goesmann, Linda Falgenhauer, Torsten Hain, Can Imirzalioglu, Stephen E. Mshana, Jörg Overmann, Trinad Chakraborty

**Affiliations:** 1 Institute of Medical Microbiology, German Centre of Infection Research, Site Giessen-Marburg-Langen, Justus-Liebig-University Giessen, Giessen, Germany; 2 School of Biotechnology, KIIT University, Bhubaneswar, Odisha India; 3 Leibniz Institute DSMZ-German Collection of Microorganisms and Cell Cultures, and German Centre of Infection Research (DZIF), Partner Site Hannover-Braunschweig, Inhoffenstraße 7B, 38124 Braunschweig, Germany; 4 Department of Chemistry, Biochemistry and LOEWE-Center for Synthetic Microbiology, Philipps-University Marburg, Marburg, Germany; 5 Bioinformatics and Systems Biology, Justus Liebig University, Giessen, Germany; 6 Department of Microbiology, Weill Bugando School Medicine, P.O. Box, 1464 Mwanza, Tanzania

## Abstract

Nosocomial pathogens can cause life-threatening infections in neonates and immunocompromised patients. *E. bugandensis* (EB-247) is a recently described species of *Enterobacter*, associated with neonatal sepsis. Here we demonstrate that the extended spectrum ß-lactam (ESBL) producing isolate EB-247 is highly virulent in both *Galleria mellonella* and mouse models of infection. Infection studies in a streptomycin-treated mouse model showed that EB-247 is as efficient as *Salmonella* Typhimurium in inducing systemic infection and release of proinflammatory cytokines. Sequencing and analysis of the complete genome and plasmid revealed that virulence properties are associated with the chromosome, while antibiotic-resistance genes are exclusively present on a 299 kb IncHI plasmid. EB-247 grew in high concentrations of human serum indicating septicemic potential. Using whole genome-based transcriptome analysis we found 7% of the genome was mobilized for growth in serum. Upregulated genes include those involved in the iron uptake and storage as well as metabolism. The lasso peptide microcin J25 (MccJ25), an inhibitor of iron-uptake and RNA polymerase activity, inhibited EB-247 growth. Our studies indicate that *Enterobacter bugandensis* is a highly pathogenic species of the genus *Enterobacter*. Further studies on the colonization and virulence potential of *E. bugandensis* and its association with septicemic infection is now warranted.

## Introduction

*Enterobacter* species are widely encountered in nature and are often part of the intestinal microbiota of both humans and animals. *Enterobacter* infections can include bacteremia, lower respiratory tract infections, skin and soft-tissue infections, urinary tract infections (UTIs), endocarditis, intra-abdominal infections, septic arthritis, osteomyelitis, CNS and ophthalmic- infections^1^. They are also of clinical importance as nosocomial opportunistic pathogens, being increasingly associated with neonatal sepsis and infections in immunocompromised hosts leading to bloodstream infections^2^. Species of the genus *Enterobacter* are ubiquitous and are very well adapted to the environment in healthcare institutions^3^. They can survive on skin and dry surfaces as well as replicate in contaminated fluids^4^. Numerous outbreaks have been described, particularly infections associated with contaminated enteral feedings, humidifiers and respiratory therapy equipment^1^. Currently *E. cloacae* and *E. hormaechei* are most frequent members of this species isolated from intensive care patients^1,5^.

Treatment of infections with *Enterobacter* spp. is notoriously difficult and broad resistance to third generation cephalosporins, penicillin and quinolones is an increasing problem^3^. As *Enterobacter* spp. are multi-drug resistant, a combination of antibiotics are prescribed simultaneously for serious infections^6^. Despite the relevance of this bacterial species in nosocomial infections, the pathogenic mechanisms and factors involved in causing disease are not understood. This is in part due to the lack of appropriate models of infection required to assess virulence properties.

Gram-negative bacteria causing septic infections express a range of virulence and fitness factors such as pili, flagella, outer membrane proteins, capsules, exotoxins and endotoxins, iron acquisition systems and niche-specific metabolic pathways^7^. Often, the ability of pathogenic bacteria to overcome oxidative stress and serum-mediated bactericidal activity of the host are characteristic virulence traits^8,9^. Studies with other enterobacterial species, in particular, *Escherichia coli*, have uncovered properties that contribute to bacterial survival in adverse host environments^10^. However, this has been much less studied in *Enterobacter* spp. so far.

We used a multi-level systems approach to determine the pathogenic potential of the newly described species *E. bugandensis*^11^. A representative isolate EB-247, which is the type strain of *E. bugandensis*, obtained from a blood sample of an infected neonate, is a facultative aerobic bacterium that grows optimally at 37 °C^12^. EB-247 is highly motile, capsular and can grow in environments of up to 9% NaCl or 90% (v/v) of human serum^11^. EB-247 is resistant to ampicillin, amoxicillin/clavulanic acid, piperacillin/sulbactam, piperacillin-tazobactam, cefalotin, cefuroxime, cefuroxime-axetil, cefoxitin, cefpodoxime, cefotaxime, ceftazidime, gentamicin, tobramycin, ciprofloxacin, norfloxacin, tetracycline and trimethoprim/sulphamethoxazol^12^. We assessed the pathogenic potential of EB-247 in an insect and a mouse model and compared this to those ofother *Enterobacter* species, known to cause infection. To gain deeper knowledge into the mechanisms mediating antibiotic resistance, as well as fitness and virulence properties, the genome of EB-247 was sequenced to completion. RNA-sequencing (RNA-Seq) based transcriptomic analysis was used to investigate global changes in gene expression during growth in human serum. Based on the data obtained, we examined the ability of an inhibitor of iron uptake in restricting growth as an alternative to antibiotic-based treatment regimens.

## Results

### General characteristics of the genome

The closed genome of *E*. *bugandensis* consists of a circular chromosome of 4,717,613 base pairs, comprising 4,355 genes with a G + C content of 56%. Alignment of the genome to other available *E. cloacae* genomes showed that there are several species-specific genomic regions. We detected a total number of 16 GIs using SIGI-HMM. Functional annotation of genes carried by these predicted GIs are shown in Supplementary File S1. A large number of GIs encode products involved in the biosynthesis of capsule polysaccharides, efflux pumps and toxin (colicin E2) production indicating that these elements contribute to survival in the infected host.

### Virulence mechanisms revealed by genome analysis

Whole genome analysis of EB-247 revealed the presence of genes involved in capsule formation in two different chromosomal locations. We found 72 homologs of virulence genes associated with EB-247 (Table S1). The strain contains an intact type VI secretion system (T6SS) and the biosynthetic machinery for colicin E2 production, a peptide known for its antagonistic activities towards *E. coli*. The genome of EB-247 also harbors many genes involved in iron acquisition, storage and metabolism, serum-resistance, heavy metal resistance as well as genes encoding adhesins and those required for biofilm formation.

### Plasmid-encoded genes

Strain EB-247 harbors an IncHI2 plasmid (pEB-247) of 298,984 bp in size. Resistance genes were transferable to *Escherichia coli* J53 when using cefotaxime as antibiotic and only at 20 °C but not at 37 °C (conjugation efficiency at 20 °C = 3.14*10^−4^). The plasmid displayed features that are commonly present on other IncHI2 plasmids (arsenate resistance, tellurite resistance, a transfer region split into two sections, and two different replication genes). It carried a number of antibiotic resistance genes (*bla*_CTX-M-15_*, bla*_TEM-1_*, bla*_OXA-1_*, tetA/R, strA/B, aac*(*6′*)*Ib-cr, qnrB1, aacC3, catA1, catB3, aadA1, sul2, tmrB, dfrA14*) indicating that its multi-drug resistance phenotype is exclusively due to plasmid-encoded genes (Fig. S1, Table S2).

Database search using blastn (http://blast.ncbi.nlm.nih.gov) with pEB247 as the query revealed strong homology with two other plasmids in the database (IncHI2, database no. LN794248, isolated from a Kenyan patient; pSTm-A54650, database no. LK056646.1, isolated in Malawi, called pSTm-BTCR in the initial publication^13^. pSTm-A54650 was isolated from a *Salmonella enterica* serovar Typhimurium strain causing a bloodstream infection leading to the death of the patient^13^.

All three plasmids were isolated in East-Africa, but are from different species (*Salmonella enterica* or *Enterobacter*). Compared to pEB247, the plasmid IncHI2 showed 47 SNPs whereas pSTm-A54650 harbored 51 SNPs (File S2). The comparison of pEB247, IncHI2 and pSTm-A54650 is presented in Fig. S1. The existence of three almost identical, multidrug resistance plasmids from different locations in East Africa suggests that this is a wide-spread and probably highly successful plasmid in this region. As this plasmid is not only restricted to *Enterobacter* spp., but also found in *Salmonella*, its transfer and presence in other Enterobacteriaceae spp. is inevitable.

### Virulence assessment of EB-247

**Figure 1:**
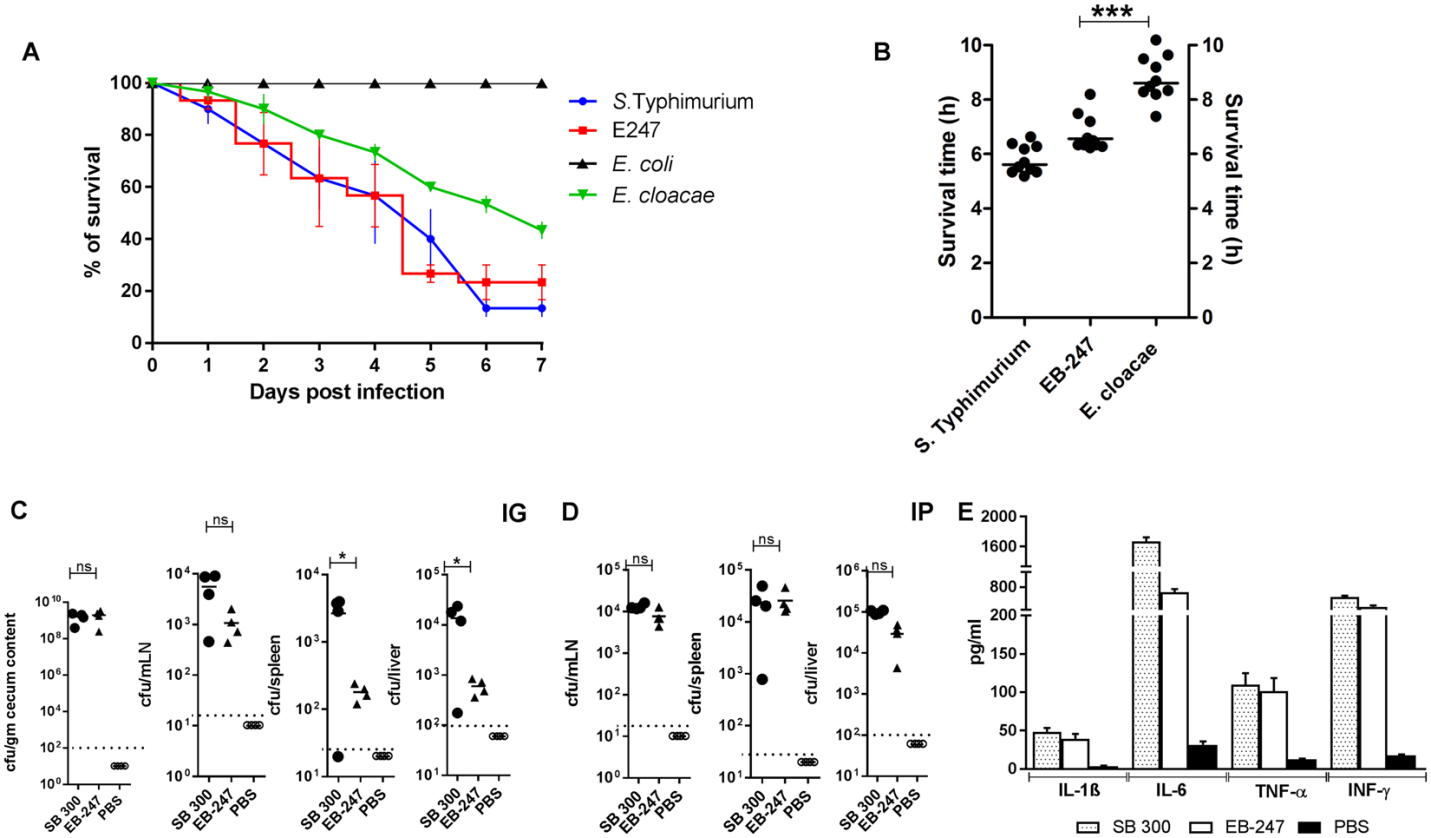
Virulence assessment of EB-247 in insect and mouse model. (**A**) Survival rates of *G*. *mellonella* after the infection of different bacterial pathogens. *G. mellonella* were infected and observed until 7 days post infection. For each group 10 larvae were infected with ~10^**2**^bacterial cfu and incubated at 37 °C. The data shown are obtained from the three independent experiments. (**B**) *G*. *mellonella* response to high dose infection. To observe the dose dependent virulence of EB-247, ~10^4^ cfu were infected to *G*. *mellonella* (n = 10) and monitored for several hours post infection. (**C**) Intra-gastric (IG) infection of BALB/c mice (n = 4) with EB-247 and SB 300. (**D**) Intra-peritoneal (IP) infection of BALB/c (n = 4) with EB-247 and SB 300. For both the IG and IP route of infection, the mice were infected with 10^5^ and 10^2^ cfu of EB-247 and SB 300 respectively. For both routes of infection, the mice were sacrificed on day 2 post infection and the bacterial colonization was assessed in different organs. (**E**) Cytokines measurement of mice following intra-peritoneal infection. The blood samples from individual mice infected with IP were collected by retro orbital bleeding and cytokines were estimated using a bead-based immunoassay technique. The dotted lines in the graphs indicate minimum detection limits. ns, not significant; *statistically significant (p < 0.05, t test).

To examine the virulence properties of EB-247, we used the larvae of wax moth *G. mellonella* and BALB/c mice as infection models, respectively^14^. Larvae were injected with 10^2^ cfu of EB-247, *Enterobacter cloacae* ATCC 13047 (*E. cloacae*), *Escherichia coli* MG1655 (*E. coli*) and *Salmonella* Typhimurium ATCC 14028 (S. Typhimurium) and monitored over 7 days for survival. EB-247, *E. cloacae*, *S*. Typhimurium and *E. coli* showed overall survival rates of 77%, 56%, 87% and 100% respectively (Fig. 1A). Injection of 10^4^ cfu of strain EB-247 led to rapid larval death within 6 h as compared to 5 h with S. Typhimurium and 9 h for *E. cloacae* (Fig. 1B).

We next assessed the virulence properties of EB-247 in BALB/c mice. For these studies, we used two different routes of infection: intra-gastric (IG) as well as intra-peritoneal (IP). Animals that were IG infected were pretreated with 50 mg of streptomycin in order to overcome the competition of gut luminal microbiota^15^. Mice were infected with EB-247 and *S*. Typhimurium (SB300) using intra gastric and intra peritoneal routes. Another group of mice was treated with PBS and was used as a control throughout the experiment. To evaluate the colonization potential of EB-247 in gut lumen and systemic sites, the animals were sacrificed 48 h post infection. Both EB-247 and SB300 exhibited comparable levels of colonization in the cecum (Fig. 1C). Like SB300, EB-247 also found to colonize the mLN whereas colonization of EB-247 in spleen and lever was significantly lower in comparison to SB300 when infected orally (Fig. 1C). At the same time, EB-247 found to colonize in the systemic sites when infected intraperitoneal (Fig. 1D). We also analyzed the cytokine response in the mice infected with EB-247 and SB300 and compared with the control treated with PBS. We measured the serum level of cytokines like IL-1, IL-6, TNF-α and INF-γ as they are known to contribute to septic shock caused by Gram negative organisms^16–18^. The level of cytokines detected in the mice infected with EB-247 was comparable to that of mice infected with SB300 (Fig. 1E). Taken together, these data indicate that the EB-247 is capable to infect, colonize and to induce inflammatory response in the host.

### EB-247 harbors very long O-antigens that induce cytokine production *in vitro*

**Figure 2:**
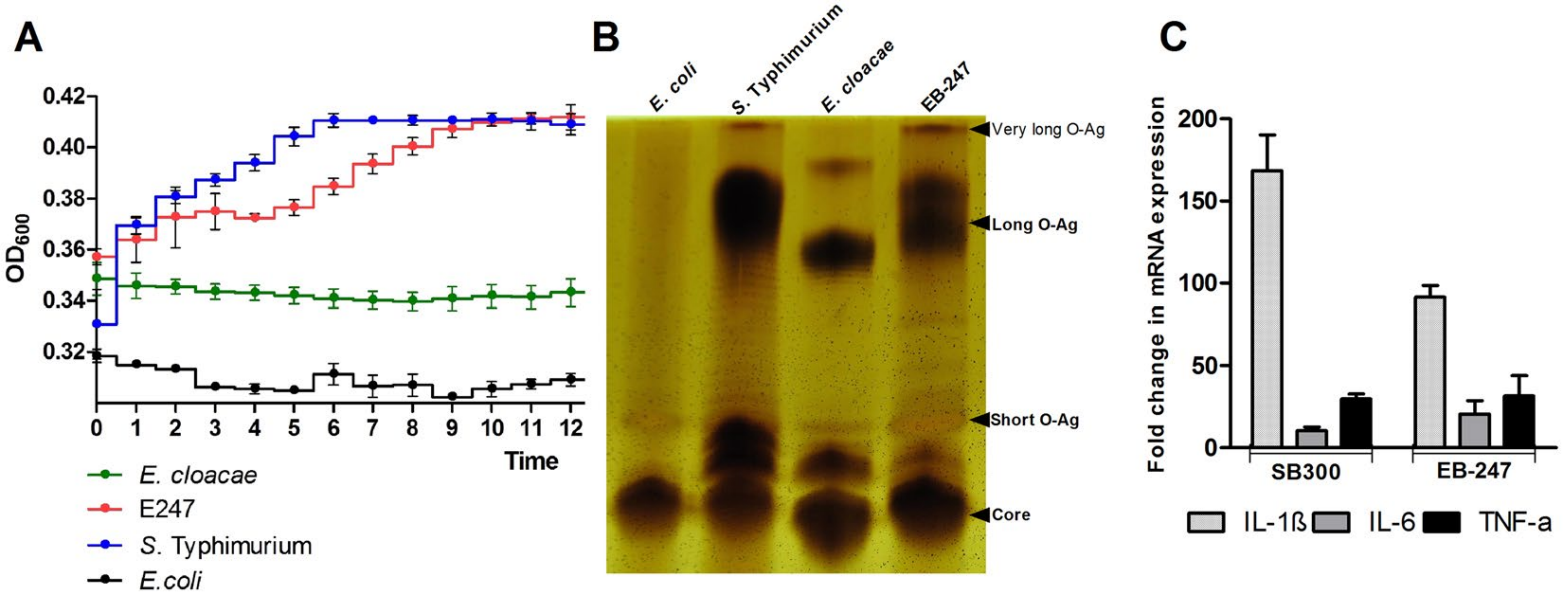
Growth of EB-247 in human serum and physical characterization of EB-247 O-antigens and their role in cytokine induction *in vitro*. (**A**) The growth pattern of EB-247 in 50% human serum. Five µL from overnight cultures of individual strains were suspended in human serum and incubated at 37 °C for 22 h in a 96-well microtiter plate. The OD_600_ was measured in every hour by the incubator cum microtiter plate reader. Data represented means ± SD of measurements performed in triplicate. (**B**) Characterization of EB-247 lipopolysaccharide (LPS). The LPS of individual strains were extracted by using the hot phenol water method and electrophoresed in a Tris-Glycine polyacrylamide gel. Further, the gel was stained by using a conventional silver staining protocol. (**C**) Estimation of fold change in the mRNA expression of IL-1, IL-6 and TNF-α upon LPS stimulation in BMDM cells. The cells were stimulated with 0.5 µg/mL of LPS isolated from EB-247 and SB 300. The BMDM cells were harvested 4 h post stimulation and the cDNA was prepared from the isolated total RNA. In addition, quantitative gene expression was measured using qRT PCR. The data represents the mean with standard deviation of three individual experiments with three replicates of each.

As EB-247 showed its ability to survive and disseminate in the vertebrate host, we analyzed the serum resistance capacity of EB-247. We found EB-247 to be highly resistant to the killing properties of human serum (Fig. 2A). As serum resistance is associated with the length and composition of LPS, we isolated the LPS of EB-247 and compared it to that of *S*. Typhimurium, *E*. *cloacae* and *E*. *coli*. We detected O-antigens with high molecular weights for EB-247 and *S*. Typhimurium, while the O-antigen for *E*. *cloacae* was significantly shorter (Fig. 2B). We also analyzed the cytokine induction in mouse bone marrow derived macrophages by treating with LPS isolated from EB-247 and *S*. Typhimurium (Fig. 2C). Treatment of mouse bone marrow-derived macrophages with LPS extracted from EB-247 induced the expression of the mRNAs encoding IL-1ß, IL-6 and TNF-α, thereby revealing the potential of EB-247 to induce inflammation.

### Comprehensive global changes in the EB-247 transcriptome during growth in human serum

**Figure 3:**
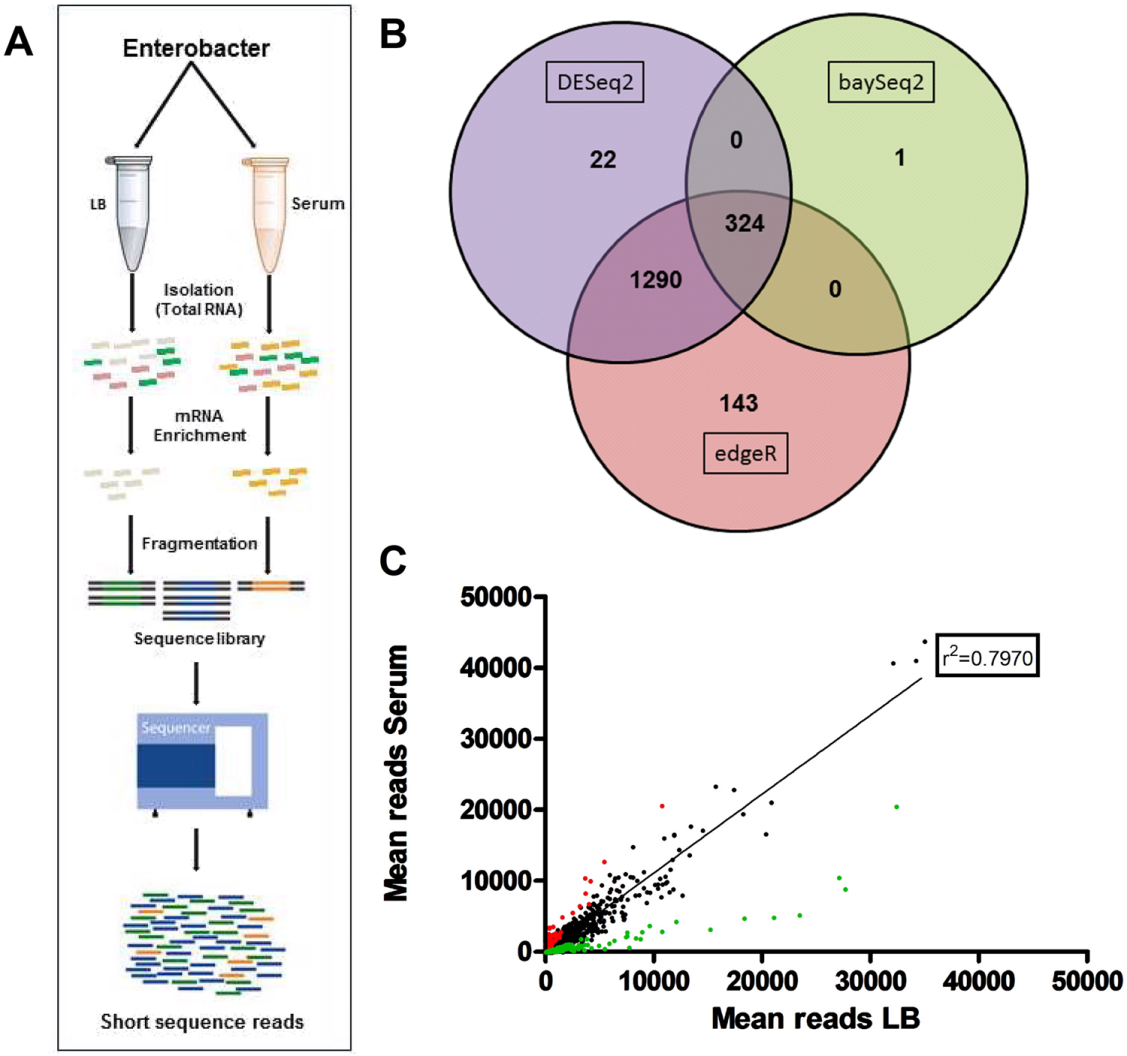
Whole genome transcriptome analysis using an RNA-Seq approach. (**A**) Experimental design of the RNA-seq approach. Schematic illustration of the steps that are employed during the sequencing procedure. (**B**) Differential expression analysis of normalized reads. After normalization, the sequence reads were analyzed by using the DESeq2, baySeq and edgeR packages. Individual packages were run independently and genes that were common according to all three different analyses were considered as differentially regulated. (**C**) Correlation between the average read counts of individual genes regulated in both LB and serum. Red and green dots represent the individual genes that are up and down regulated respectively, whereas the black dots represent the genes that are not differentially regulated. Each dot represents the paired coordinate value between the mean numbers of reads of individual genes obtained from experiments from three biological repeats. The correlation coefficient value was determined by using the approach ‘Goodness of Fit’ of GraphPad Prism V5.

**Figure 4:**
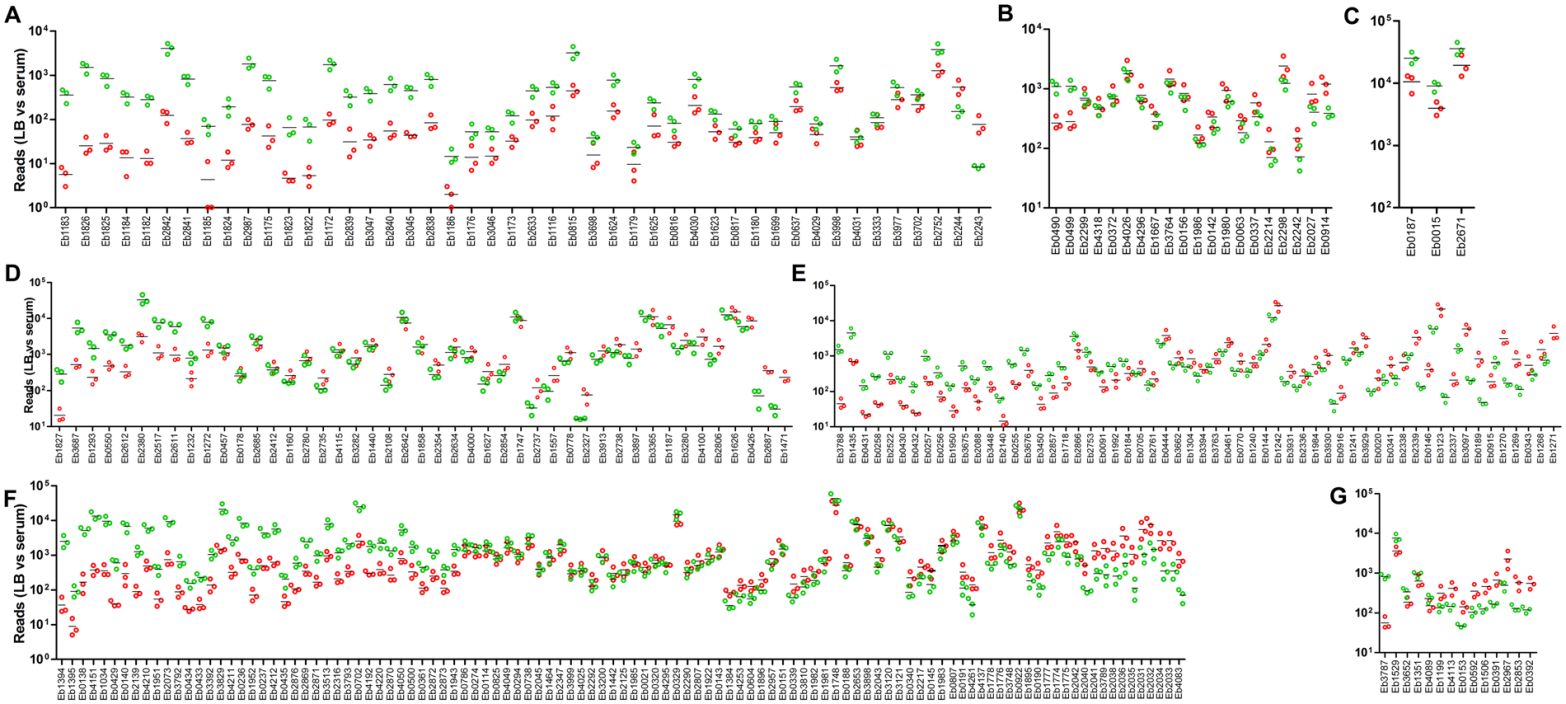
Transcriptional profile of differentially regulated genes grouped according to their most likely functions in EB-247 adaptation to and survival in human serum. (**A)** Iron uptake, (**B**) regulatory function, (**C**) chaperons, (**D**) others, (**E**) transport and binding, (**F**) energy metabolism, and (**G**) membrane proteins. The circles represent the number of reads mapped for a single gene. Green color represents the reads obtained from the sample grown in serum, while red represents reads obtained from EB-247 grown in LB. The data shown is the result of three independent biological experiments.

The RNA-seq-based transcriptomic analysis was performed to examine the differential response of the transcriptome of bacteria grown in serum as compared to growth in broth medium (Fig. 3A–C). Of the 4355 genes from the EB-247 genome, 325 genes displayed statistically significant changes in mRNA level that were more than 5-fold (p-value 0.05), of which 141 were up and 184 genes were down regulated respectively (Fig. 4A–G). These genes were grouped according to their functions and ranked according to the extent of change in expression. Up-regulated genes involves those responsible for iron uptake, transport and binding, energy metabolism, chaperones, membrane proteins, hypothetical genes and other miscellaneous genes. More specifically 46 genes contributing to iron uptake and 58 genes contributing to metabolite transport were observed to be differentially regulated (File S3). We also found 20 regulatory and 45 miscellaneous genes that were differentially regulated.

### Metabolic adaptation of EB-247 to human serum

**Figure 5:**
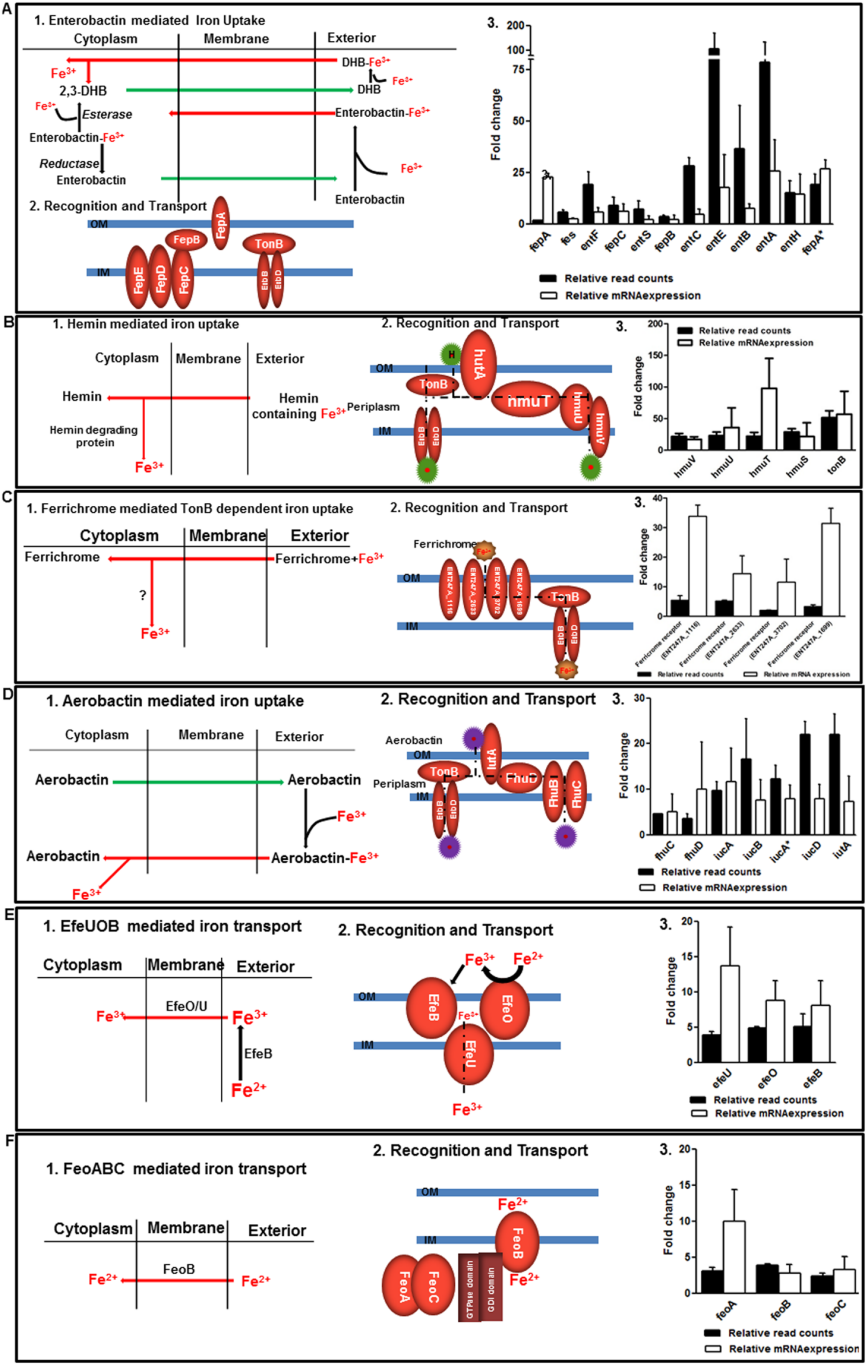
Schematic representation of mechanisms associated with EB-247 iron acquisition and differential expression analysis of genes. (**A**) Enterobactin mediated iron uptake. (**B**) Hemin mediated iron uptake. (**C**) Ferrichrome mediated iron uptake. (**D**) Aerobactin mediated iron uptake. (**E**) EfeUOB mediated iron transport. (**F**) FeoABC mediated iron transport.

The adaptation of EB-247 to human serum involves the expression of a large number of proteins involved in transport processes and energy metabolic pathways. As the availability of iron is essential for the intracellular growth of pathogenic bacteria, we analyzed the mechanisms that are associated with iron acquisition in EB-247. We detected six different iron acquisition systems that were induced during incubation in serum. These systems include genes encoding the siderophores like enterobactin (*ent*), aerobactin (*iuc*) and ferrichrome (*fhu*), their cognate receptors and uptake proteins, the Fe^+2^ uptake systems *feo* and *efe*, as well as hemin uptake by a putative receptor protein (*hut*A). Iron uptake is facilitated by TonB-ExbBD complex, an ABC transport system that is required to generate the proton motive force to promote entry of this outer membrane iron-receptor complex. Genes encoding this complex were also strongly upregulated (Fig. 5A–F). We observed increased expression of genes involved in the uptake of tricarboxylates (*tct*A) suggesting intermediate metabolites of the tricarboxylic acid (TCA) cycle as important carbon and energy sources during growth in serum (File S3E). In addition, genes involved in metabolic conversion, e.g. those encoding malate synthase and iso-citrate lyase were found to be highly expressed. Genes encoding transporters for sulfate, amino acids, putrescine, sodium and magnesium were also up-regulated. Evidence for increased gluco- and keto- neogenesis was suggested by the upregulation of biosynthetic pathways involved in amino acids such as cysteine, methionine, arginine, threonine and histidine. Genes promoting anaerobic growth using ascorbic acid as carbon source were also upregulated.

### Identification of additional genes that contribute to EB-247 serum resistance

**Figure 6:**
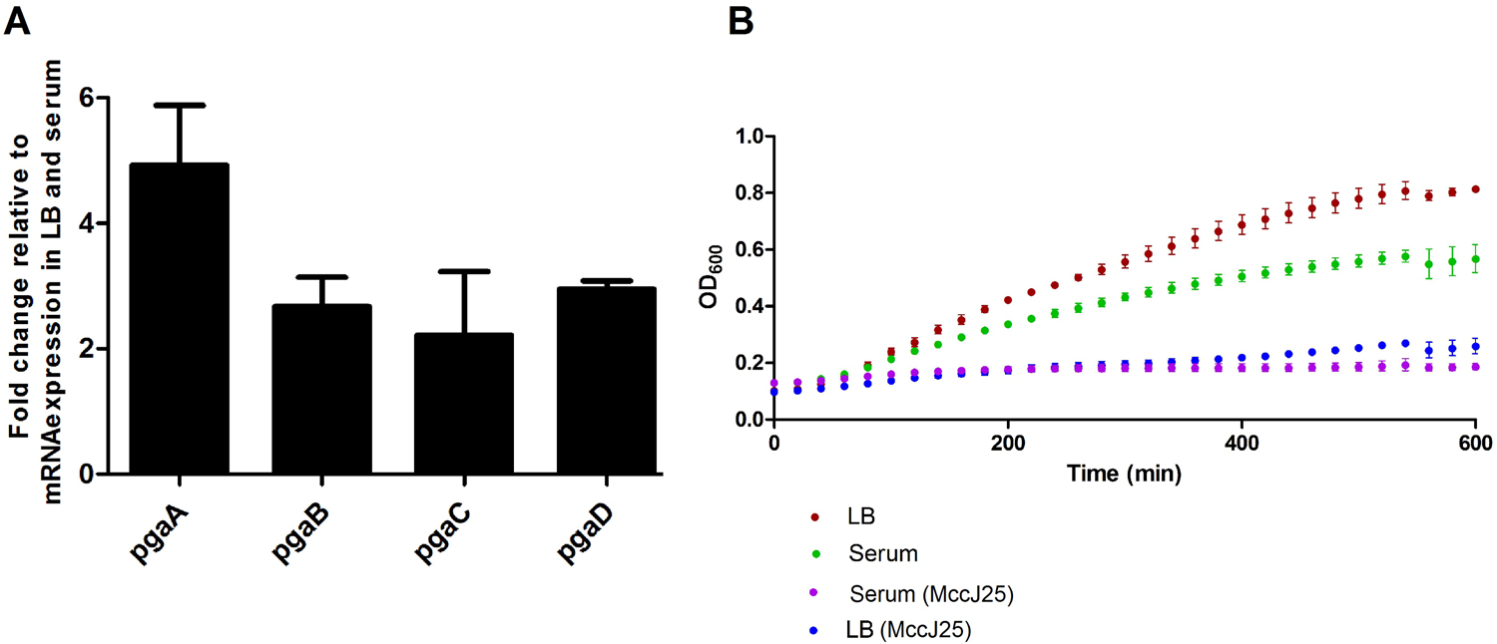
Differential expression of the *pga* operon in response to human serum and growth inhibition of EB-247 by MccJ25. (**A**) Relative fold-change in the mRNA expression level of the *pga* operon. EB-247 was treated with human serum and the total RNA was isolated. The differential expression was examined using qRT PCR. Data is presented as mean ± standard deviation of three individual experiments with three replicates each. (**B**) Growth curve of EB-247 in presence of MccJ25. EB-247 was incubated in 25% serum and LB with a concentration of 0.6 µg/mL of MccJ25. The growth was monitored over 600 min. The data for the growth curves are the representatives of three individual experiments.

We observed additional genes with enhanced expression in response to serum, which probably contribute to the serum survival of EB-247. Genes encoding for proteins like PiuB, bacterioferritin-associated ferredoxin and ferritin showed considerably enhanced expression in serum (File S3A). These groups of proteins are associated with iron uptake and storage in a non-toxic form in bacteria indicating EB-247 iron storage potential along with acquisition. Notably, the *pgaABCD* operon that is involved in biofilm formation was upregulated during growth in serum (Fig. 6A).

### EB-247 is sensitive to MccJ25

As our studies showed a strong expression of the TonB-dependent ferrichrome uptake machinery, we used the lasso peptide MccJ25 that targets this uptake machinery for growth inhibition studies. Indeed, incubation of EB-247 in presence of MccJ25 at a concentration of 0.25 nmol/µL, inhibited growth in both LB as well as in medium with 25% serum (Fig. 6B) indicating lasso peptide MccJ25 as a potential therapeutic candidate to treat MDR strains.

## Discussion

In this study, we characterized and determined the complete genome of a representative strain of new *Enterobacter* species, i.e. *E. bugandensis* EB-247 isolated from a pediatric intensive care unit. We assessed the virulence properties of EB-247 *in vivo* using invertebrate and vertebrate models. Infection experiments with *G. mellonella* demonstrated that EB-247 was more pathogenic than *E*. *cloacae* ATCC 13047, which is the most virulent *E*. *cloacae* strain known, and was as pathogenic as *S*. Typhimurium in this invertebrate model. In an oral model of mouse infection, EB-247 was able to colonize the gut lumen and mLN. This indicates the ability of EB-247 to cross the epithelial barrier and colonize the mLN which is in line with the previous finding that the luminal sampling of enteric pathogens is independent of their active invasion^19^. Recently it was demonstrated that *S*. Typhimurium is uniquely capable of utilizing galactarate and glucarate generated by host-mediated oxidation of glucose and galactose^20^. As utilization of these oxidized forms require both specific uptake and metabolic genes, we searched the genome of *E. bugandensis* for operons encoding these metabolic pathways. Genes required for both glucarate and galactarate were present in *E. bugandensis* (Fig. S2). Thus, as for *S*. Typhimurium, post-antibiotic expansion of *E. bugandensis* may be a significant factor in exacerbation of disease. The isolate actively colonized the spleen and liver when delivered directly through intraperitoneal into the host and induced a strong host inflammatory response. We identified both long and very long O-antigens as potential mediators of cytokine induction.

The ability to survive in different organs suggested haematogenous spread and an ability to grow in blood is an important survival property. We found that EB-247 is capable to grow in very high concentrations of human serum. An RNA-seq approach identified genes differentially expressed during growth of EB-247 in serum and permitted the identification of those genes involved in metabolic adaptations required for growth in blood. Genes comprising of 7% of the total genome of EB-247 were mobilized to enable growth in serum. They include genes for the uptake of carbon sources (citrate, maltose, lactate and glycolate) during aerobic growth and specific polyamines such as putrescine, that is also a vital contributor to pathogen fitness and virulence^21,22^. To enhance its metabolic adaptation, EB-247 increased the production of enzymes of the glyoxylate shunt that serves as an alternative to the tricarboxylic acid cycle for the essential production of acetate and fatty acid metabolism^23^. Upregulation of the glyoxylate shunt has previously been implicated in mediating protection of bacteria to oxidative stress, antibiotic stress, and host infection^24^. Up-regulation of ascorbic acid uptake associated genes indicates the ability of EB-247 to grow in anaerobic conditions.

Other genes upregulated in serum include those required for nucleotide biosynthesis, ABC transporters, amino acid biosynthesis and in particular, iron acquisition systems. A few genes included in the group of ‘energy metabolism’ as responsible for amino acid biosynthesis, were differentially upregulated indicating that amino acids are an important nutrient source for EB-247 during growth in serum. To investigate metabolic pathways of interest, we used KEGG-based functional annotation to assign 2,856 out of the 4,355 genes and develop a metabolic pathway map (Fig. S3). Apart from housekeeping functions, the metabolic response involved alterations in lipid, carbohydrate and nucleotide metabolism, as well as cofactors, vitamins and amino acids. These data suggest that *E. bugandensis* uses various host metabolites for survival inside the host.

As iron availability is limited in serum, a large portion of the adaptive response involved in iron uptake mechanisms and metabolism. We found that EB-247 employs four siderophore dependent and two independent mechanisms to scavenge iron from the serum, which includes enterobactin, aerobactin, hemin, ferrichrome, Efe and Feo dependent iron uptake systems as well as ferritin and bacterioferritin are significantly up regulated. These data suggest that the scavenged ferric (Fe^3+^) iron is probably dissociated from the chelate and reduced to the ferrous (Fe^2+^) form, which is stored intracellularly during its growth in iron-limiting conditions.

As EB-247 is a multidrug-resistant isolate, we explored the use of the lasso peptide MccJ25 that was previously shown to exhibit antimicrobial activities against multiple bacterial species of the family of *Enterobacteriaceae*^25^. MccJ25 first enters the bacterial periplasm by hijacking the iron siderophore receptor FhuA and further inhibits bacterial growth by blocking the NTP uptake channel of the bacterial RNA polymerase. In this study, we demonstrated that MccJ25 is a potent inhibitor of EB-247 growth in serum as well as in LB.

In conclusion, our study demonstrates that *E. bugandensis* is highly virulent and possibly the most pathogenic species of the genus *Enterobacter* currently known. The finding that all antibiotic resistance genes are encoded on a widely occurring transmissible plasmid is a reminder of the ease with which pathogenic bacteria may acquire a MDR phenotype with devastating consequences during outbreaks. Our data suggest that *E. bugandensis* has well-developed systems enabling bacterial growth following infection of the host and employs many genes involved in iron acquisition, storage and its metabolism. We targeted the siderophore receptor FhuA which is an important component of this response using the specific lasso peptide microcin MccJ25 and show that it has a bactericidal effect on *E. bugandensis* E247. Our studies encourage further exploration of the use of lasso peptides in treating MDR infection. Finally, detection of *E. bugandensis* in different settings worldwide and more studies on its virulence and colonization potential are now warranted.

## Materials and Methods

### Ethics statement

All animal experiments were performed in strict accordance with the guidelines laid by CPCSEA, Ministry of Environment, Forest and climate change, India. This study was approved by the Institutional Animal Ethics Committee (IAEC) of the School of Biotechnology, KIIT University with approval number KSBT/IAEC/2015/MEET-1/A1. All efforts were made to minimize suffering of animals during experimentation. For human serum based experiments, all blood samples were collected from the 10 healthy adult individuals following written informed consent at Institute of Medical Microbiology, Justus-Liebig-University Giessen, Germany.

### Bacterial strains and growth conditions

Bacterial strains listed in Table 1 were grown in LB agar and broth at 37 °C. Respective antibiotics like ampicillin (100 µg/mL), kanamycin (50 µg/mL), chloramphenicol (50 µg/mL) and cefotaxime (20 µg/mL) were used to select the strains. For *in vivo* infection experiments the bacteria were grown for 12 h at 37 °C in LB broth, diluted 1:20 in fresh LB medium and sub-cultured for 4 h under mild aeration. Bacteria grown to an OD of ~0.6 were pelleted, washed with ice cold phosphate buffered saline (PBS) and resuspended in 50 μl cold PBS was used for different routes of infection.

**Table 1:** Strains used for this study.

Sr. No.	Strain Name	Selective Antibiotic	Source
1	*Enterobacter bugandensis* EB-247	Cefotaxime	This study
2	*Enterobacter cloacae* ATCC 13047	Ampicillin	DSMZ
3	*Salmonella* Typhimurium ATCC 14028	—	ATCC
4	*Salmonella* Typhimurium (SB 300)	Streptomycin	Pati *et al*. 2012
5	*Escherichia coli* MG1655	—	ATCC

### Whole genome sequencing

For whole genome sequencing, genomic DNA was isolated from the cultures grown in LB medium at 37 °C for 12 h. The DNA was isolated using a Purelink™ DNeasy Kit (Invitrogen, Germany) according to the manufacturer’s instructions and sequenced using Illumina MiSeq as well as PacBio. For Illumina MiSeq, DNA sequencing libraries were prepared using the Nextera® XT kit (Illumina, USA) and paired-end reads 2 × 300 were sequenced. For PacBio, SMRTbell™ template library was prepared using P4 Chemistry generating 4,053 pre-filtered reads with an average read length of 5,063 bp. The assembled genome was annotated by GenDB^26^ and the ‘dnaA’ gene was adjusted as first gene. The complete closed chromosome, plasmids and all the raw reads are deposited under the project with accession number ERS726104. For plasmids, the core genome single nucleotide polymorphisms (SNPs) was identified using ParSnp^27^. The virulence and antibiotic resistance genes were identified by BLASTp against the VFDB and CARD respectively^28,29^.

### Galleria mellonella infection assay

*Galleria mellonella* larvae were reared on an artificial diet (22% maize meal, 22% wheat germ, 11% dry yeast, 17.5% bees wax, 11% honey and 11% glycerin) at 32 °C in incubators before infection. The matured larvae with a weight of 250–350 mg were used for the infection experiment. For infection, strains were grown in LB at 37 °C while constantly shaken at 180 rpm. Overnight grown bacterial culture was diluted 1:100 and then grown to mid exponential phase (OD_600_ = 1.0). Further, the bacteria were washed twice with 0.9% NaCl solution (w/v). Each larva was injected with 10^2^ cfu of bacteria. Post infection, the larvae were kept at 37 °C and their survival was inspected every 24 h up to 7 days.

### Mice infection experiment and cytokine estimation

The mice infection experiments were performed in specific pathogen free BALB/c mice as mentioned earlier^15^. Infections of mice were conducted in individual ventilated cages. The mice infected orally were pretreated with 50 mg of streptomycin before infecting with *S*. Typhimurium and EB-247, while the mice infected intraperitoneally were exempted from the streptomycin treatment. Four mice were placed in each group. Mice infected orally and intraperitoneally were infected with 10^5^ and 10^2^ cfu respectively. Bacterial loads in cecal content, mesenteric lymph node (mLN), liver and spleen were determined by plating homogenates on LB agar plates supplemented with appropriate antibiotic/s. The concentrations of IL-1, IL-6, TNF-α and INF-γ in the serum were detected by bead based immunoassays according to the manufacturer’s protocol (BD Cytometric Bead Array).

### Bacterial serum resistance assay

Human venous blood from 5 healthy persons was collected (age ranged from 25–40) in non-heparinized blood collection vials. The collected blood was kept undisturbed at room temperature for 30 min followed by a cold centrifugation at 3000 rpm for 5 min. Then, the serum was carefully isolated from the upper part of the tube, pooled and stored at −20 °C. For serum resistance tests, bacterial strains were grown overnight in 3 mL of LB broth with appropriate antibiotic. 5 µL of overnight bacterial culture was inoculated to 145 µL 50% human serum in LB broth prepared in a 96 well microtiter plate. The plate was further incubated in a microtiter plate reader (TECAN^TM^) and OD_600_ was measured at regular intervals of 1 h up to 22 h. A data set of 12 h was chosen to represent the growth curve for the bacteria in serum.

### Isolation of bacterial RNA and library preparation for Illumina sequencing

A single colony of EB-247 was inoculated in LB broth and grown overnight at 37 °C and 180 rpm. Further, the overnight culture was sub-cultured in fresh LB broth at a dilution of 1:20 until reaching an OD_600_ of 0.6. 1 mL of bacterial culture was centrifuged, washed with PBS and further incubated in 50% serum for 1 h at 37 °C. RNA from treated and untreated samples was isolated by enzymatic lysis using lysozyme (0.4 mg/mL), SET buffer (50 mM NaCl, 5 mM EDTA, 30 mM TrisHCl, 10% SDS), 2 µL suparase (20 U/µL), 5 µL mutanolysin (5 U/µL) and 10 µL proteinase K. The samples were vortexed and incubated at 37 °C for 30 min. RNA isolation was completed with the RNeasy Mini Kit (Qiagen) according to the manufacturer’s protocol. DNA contamination was avoided by treating the sample with RNase-free-DNase (Qiagen). The concentration and integrity of RNA was assessed by measuring the 260/280 ratio and electrophoretic analysis by employing an Agilent 2100 Bioanalyzer (Agilent Technology). Only samples having a RIN (RNA integrity) value above 9 were used for further workflow (Fig. S4).

The enrichment of mRNA was achieved by depleting the ribosomal RNA using the ribo-zero bacterial kit (Epicentre). The depleted RNA was further fragmented by incubation with metal ions (Zn^2+^) for 8 min at 94 °C using the TruSeq Stranded Total RNA Sample Prep Kit. First strand synthesis was performed using randomly primed hexamers and superscript II. In this process, actinomycin D was added in order to prevent spurious DNA amplification. Further, second strand synthesis was accomplished by replacing the RNA template strand and incorporating dUTP in place of dTTP to generate ds cDNA. cDNA fragments were end repaired, leading to the formation of blunt end cDNA. The blunt end cDNA was further adenylated at the 3′-OH end to prevent self-ligation during the adapter ligation reaction. Adapters with unique indices were ligated with the cDNA in the respective samples. Afterwards, a PCR reaction of 15 cycles was performed to enrich the adapter ligated fragments. Clean up between the steps was performed with Agencourt AMPure XP beads. Each library quality was then assessed by Agilent High Sensitivity DNA chip and the libraries were normalized and diluted according to the Illumina MiSeq protocol. Finally, all libraries were sequenced on a flow cell in a multiplexed manner using Illumina v3 chemistry.

### Analysis of transcriptome data

FastQ-files from the runs were obtained after image processing/basecalling and demultiplexing of reads performed by the Illumina MiSeq-Control Software using the RTA-versions 1.18.42 and 1.18.54 respectively. For initial manual quality control as well as for later on visualizations, those files were imported in the commercial software CLC bio genomic workbench version 7.5 and mapped. As mapping parameters length fraction and similarity of 80% together with default mismatch cost of 2, deletion and insertion cost of 3 were chosen. Mapping rates between 85 and 88% were achieved for all runs in this way. The mappings were then exported as BAM-files for further counting and differential expression analysis. The variability between each biological repeats of two different conditions was measured by evaluating the correlation coefficient (r) with the help of ‘Goodness of fit’ using GraphPad Prism V5 (Fig. S5). Data sets obtained from Illumina Miseq were additionally normalized using DESeq^30^. The read numbers obtained post normalization was used as the final read counts for the differential expression analysis. Three different expression analysis packages (DESeq2, baySeq and edgeR) were used^31,32^. The only genes that are common to all three different analyses were considered to be differentially expressed. Thereby 325 genes were found to be differentially regulated with respect to both growing conditions. These were further confirmed by Student’s t-test (p-value < 0.05) and the read numbers were manually checked using CLC Sequence Viewer.

### Preparation of cDNA and qRT-PCR analysis

The differential expression of genes shown in the transcriptome experiment was validated by two-step qRT-PCR for some of the selected genes. The primers for the genes were designed by the primer design software available in NCBI (National Center for Biotechnology Information). For analysis the transcripts were isolated from serum treated and untreated bacterial samples. cDNA synthesis was performed by priming 500 ng of total RNA using random hexamer and nonamer primers along with SuperScript II (Invitrogen). Fluorescence PCR amplification was performed by using 1 µL aliquot of a 1^st^ strand cDNA reaction with brilliant SYBER Green qPCR master mix (Qiagen) on a AppliedBiosystem cycler in a final volume of 25 µL. Specificity of all the amplicons was confirmed by gel analysis and melting curves. The experiment was performed in triplicate for three biological replicates. 16S rRNA was used as an endogenous control. Relative quantification was performed using the 2^(−ΔΔCt)^ method. All the primers used in this study was listed in Table S3.

### Preparation of Bone Marrow-Derived Macrophage (BMDM) cells, Cell stimulation by LPS and quantification of IL-1, IL-6 and TNF-α mRNA by qRT PCR

The BMDMs were obtained from cultures of bone marrow stem cells from C57BL/6 mice of either sex (8 to 12 weeks old). The bone marrow was flushed from femurs with ice cold RPMI medium supplemented with 10% FCS, 1% L-glutamic acid, 1% Na-pyruvate, 50 U/mL penicillin/streptomycin and 50 µM ßME. The marrow plugs were disrupted into single-cell suspensions by passing through 70 µm wide cut-off cell strainer. Further, the cells were centrifuged (5 min at 1500 rpm), washed and cultured at a cell density of 10^6^ nucleated cells/mL of RPMI and 10% conditioned medium. After various periods of incubation at 37 °C in 5% CO_2_, the non-adherent cells were discarded and experiments were performed on adherent cells. The adhered bone marrow cells (2 × 10^6^ cells/mL) were stimulated with 0.5 µg/mL of LPS isolated from *S*. Typhimurium and EB-247. 4 h post stimulation, the cells were harvested and the total RNA was isolated as mentioned above. The cDNA was prepared from the total RNA. 50 ng of cDNA was used as a template for the quantitative real time PCR.

### Isolation and purification of the lasso peptide MccJ25

MccJ25 was obtained by a modified protocol from a previous study^33^. In short, a 2 L baffled flasks containing 500 mL M9 minimal medium (17.1 g/L Na_2_HPO_4_∙12 H_2_O, 3 g/L KH_2_PO_4_, 0.5 g/L NaCl, 1 g/L NH_4_Cl, 1 mL/L MgSO_4_ solution (2 M), 0.2 mL/L CaCl_2_ solution (0.5 M); pH = 7.0) was supplemented with 5 mL 40% glucose solution (w/V), 250 µL chloramphenicol solution (34 mg/mL in 70% ethanol) and 1 mL M9 vitamin mix (1.0 g choline chloride, 1.0 g folic acid, 1.0 g pantothenic acid, 1.0 g nicotinamide, 2.0 g myo-inositol, 1.0 g pyridoxal hydrochloride, 1.0 g thiamine, 0.1 g riboflavin, 0.3 g disodium adenosine 5′-triphosphate, 0.2 g biotin; suspend in 300 mL ddH_2_O; slowly add 10 M NaOH until completely dissolved (final pH ~12) and sterile filter; store at 4 °C until needed). After pre-warming to 37 °C, it was inoculated 1:100 with an LB overnight culture of *E. coli* BL21 (DE3) carrying the pTUC202 plasmid^34^ and cultivated for 1 day at 37 °C. Afterwards, the cells were spun down and the supernatant was extracted by addition of 7.5 mL XAD16 suspension (100 g XAD16 resin suspended in 400 mL ddH_2_O) and slowly stirred at room temperature for 1 h. The resin was subsequently collected by filtration, washed thrice with ddH_2_O and eluted with 50 mL methanol. After removing the solvent by evaporation at 40 °C and reduced pressure, it was resuspended in 8 mL 50% methanol, centrifuged (30 min, 13000 rpm) and filtered through a 0.45 µm syringe filter. The cleared extract was then applied to two rounds of preparative HPLC employing a microbore 1100 HPLC system (Agilent) at room temperature with a flow rate of 18 mL/min and using a VP 250/21 Nucleodur C18 Htec 5 µm column (Macherey-Nagel) for separation. For the first round, a solvent system of water/0.05% formic acid (solvent A) and methanol/0.04% formic acid (solvent B) was utilized with the following gradient: Linear increase from 40% to 50% B in 5 min, followed by an additional linear increase to 95% B in another 25 min. For the second and final round of HPLC purification, a solvent system consisting of water/0.1% TFA (solvent C) and acetonitril/0.1% TFA (solvent D) was used. The subsequent gradient was employed: Initial linear increase from 25% to 45% D in 30 min, followed by a further linear increase to 95% D in additional 5 min. In this way, 9.8 mg pure MccJ25 was recovered from a 500 mL production culture.

### Lasso peptide (MccJ25) growth inhibition assay

EB-247 was cultured overnight at 37 °C in 3 mL LB broth medium. 5 µL of overnight culture was further incubated in 145 µL 25% serum in LB in presence of 0.6 µg/mL MccJ25. As control, EB-247 was also grown without serum under the same conditions. The prepared samples were placed in a 96 well microtiter plate in replicates and were allowed to grow in an incubator cum microtiter plate reader (TECAN^TM^). The growth was measured at a regular interval of 1 h with OD at 600 nm.

### Data availability

All relevant data are within the paper and its Supporting Information files. The sequencing data for this study is publicly available under the study accession number PRJEB11452 in standard databases such as NCBI or EMBL. The same can be available by using the links below; http://www.ebi.ac.uk/ena/data/search?query = PRJEB1142, https://www.ncbi.nlm.nih.gov/gquery/?term=PRJEB11452.

## Electronic supplementary material

Supplementary figures and tables

File S1 Supplementary Dataset

File S2 Supplementary Dataset

File S3
