# Supplementary material for: Enterobacter bugandensis: a novel enterobacterial species associated with severe clinical infection
Source: Sci Rep. 2018 Mar 29;8:5392. doi: 10.1038/s41598-018-23069-z (PMC5876403; doi:10.1038/s41598-018-23069-z)
Supplement: Supplementary file 1 — Supplementary figures and tables [file 41598_2018_23069_MOESM1_ESM.pdf]

Supplementary Information

Title: *Enterobacter bugandensis*: a novel enterobacterial species associated with severe clinical infection

Authors:

Niladri Bhusan Pati[1], Swapnil Prakash Doijad[1], Tilman Schultze[1], Gopala Krishna Mannala[1], Yancheng Yao[1], Sangeeta Jaiswal[2], Daniel Ryan[2], Mrutyunjay Suar[2], Konrad Gwozdzinski[1], Boyke Bunk[3], Mobarak Abu Mraheil[1], Mohamed A. Marahiel[4], Julian D Hegemann[4], Cathrin Spröer[3], Alexander Goesmann[5], Linda Falgenhauer[1], Torsten Hain[1], Can Imirzalioglu[1], Stephen E. Mshana[6], Jörg Overmann [3], Trinad Chakraborty[1]*

Affiliations:

[1]Institute of Medical Microbiology, German Centre of Infection Research, Site Giessen-Marburg-Langen, Justus-Liebig-University Giessen, Giessen, Germany.

[2]School of Biotechnology, KIIT University, Bhubaneswar, Odisha, India.

[3]Leibniz Institute DSMZ-German Collection of Microorganisms and Cell Cultures, and German Centre of Infection Research (DZIF), Partner Site Hannover-Braunschweig, Inhoffenstraße 7B, 38124 Braunschweig, Germany.

[4]Department of Chemistry, Biochemistry and LOEWE-Center for Synthetic Microbiology, Philipps-University Marburg.

[5]Bioinformatics and Systems Biology, Justus Liebig University, Giessen, Germany.

[6]Department of Microbiology, Weill Bugando School Medicine, P.O. Box 1464, Mwanza, Tanzania

Corresponding author:

Prof. Dr. Trinad Chakraborty
Institute for Medical Microbiology, German Centre for Infection Research, Giessen-Marburg-Langen site, Schubertstrasse 81, Justus-Liebig University 35394 Germany Tel: +49 641 99 41250 Fax: +49 641 9941259 Email: trinad.chakraborty@mikrobio.med.uni-giessen.de

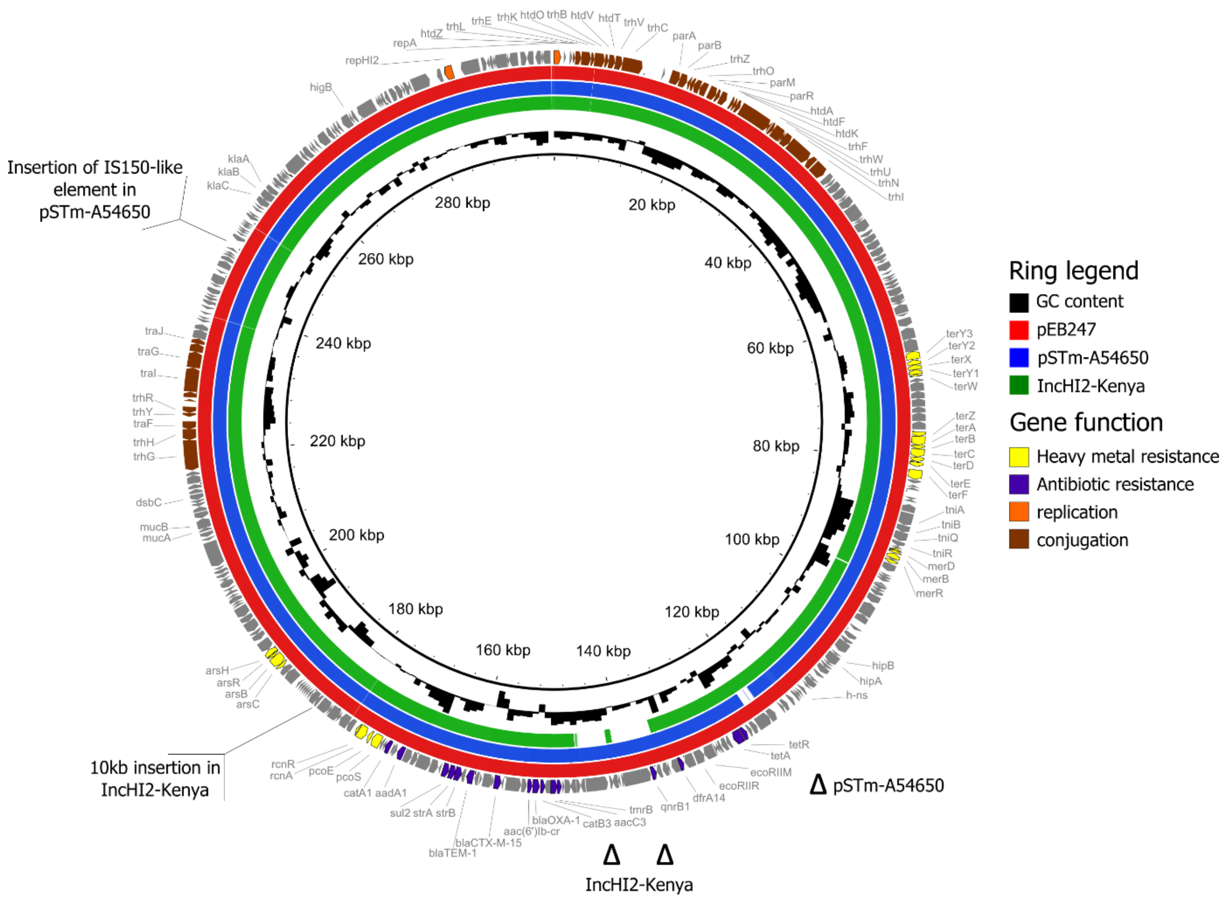

Figure S1

The details of the plasmid from EB-247 (pEB-247). The represented image shows the comparison of pEB-247 to IncHI2 and pSTm-A54650 created by G-view.

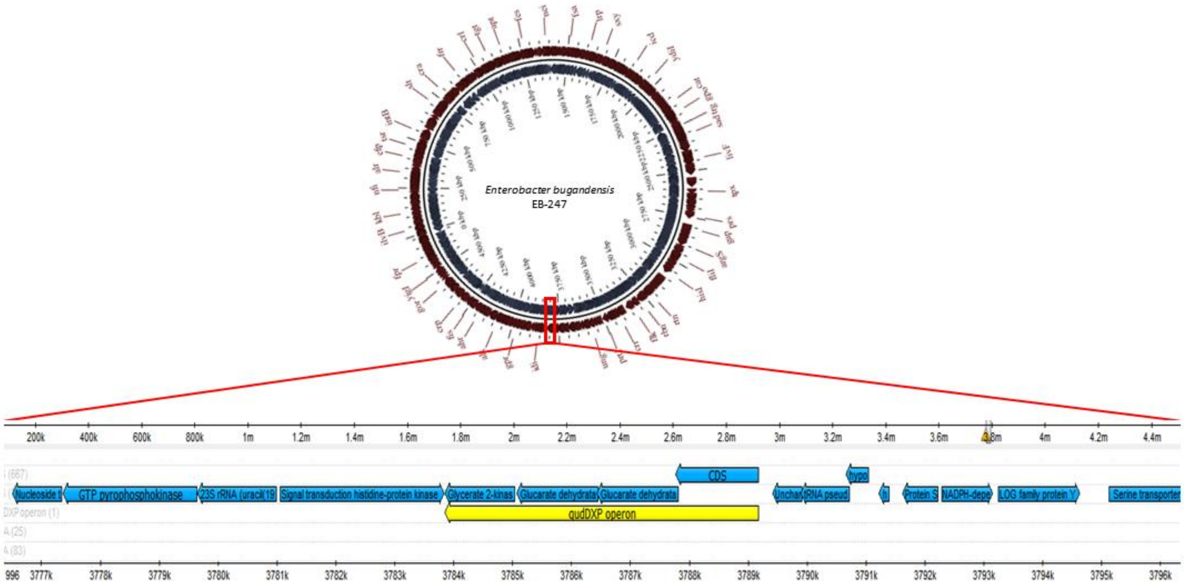

Figure S2

The *gud*DXP operon of *Enterobacter bugandensis* EB-247. The image represents the chromosomal location of the *gud*DXP operon at 3.78 Mb. The amplified region details the genetic environment of negative strand encoded *gud*DXP operon. The *gud*DXP operon enable cells to utilise glucorate and galactarate generated by host-mediated oxidation of glucose and galactose.

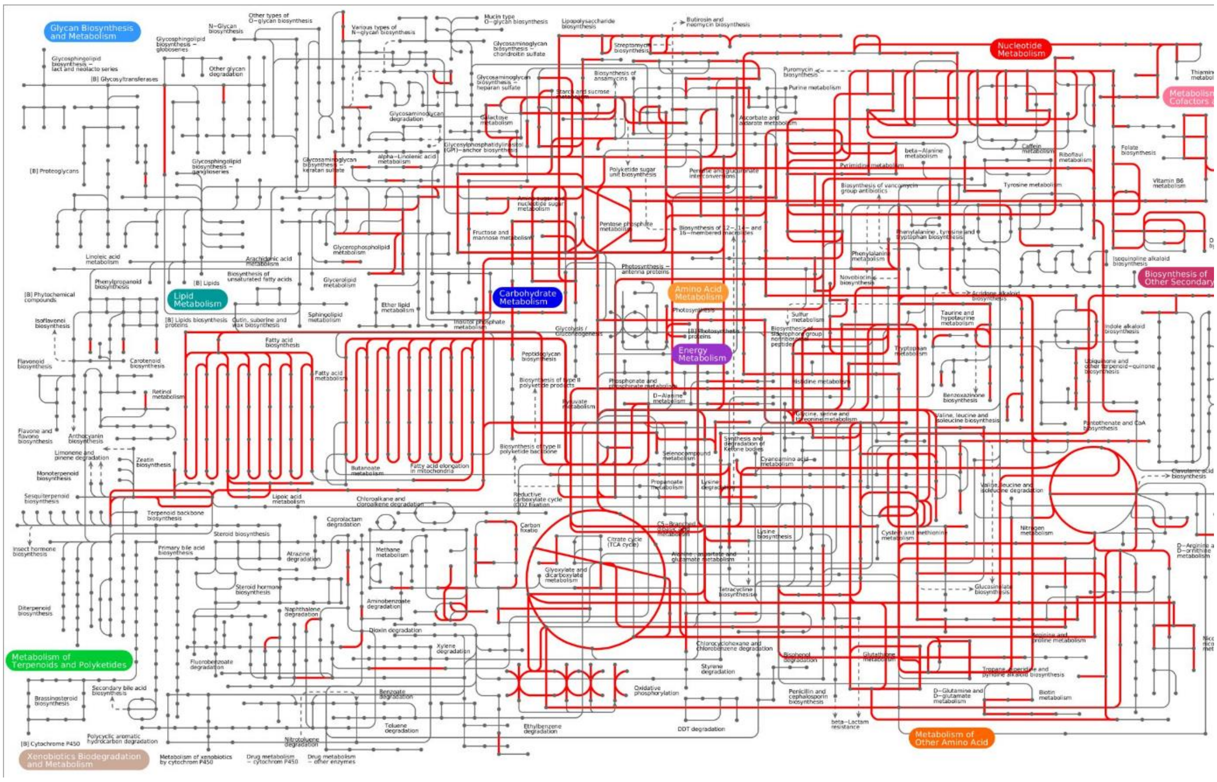

Figure S3

Global Metabolic Pathway Map of EB-247. Complete metabolism network of EB-247 is shown to indicate expressed elements of the EB-247 metabolome. Nodes in this figure are metabolic compounds. Edges are enzymatic transformations. Light grey background edges indicate KEGG pathways not encoded by the EB-247 genome. KEGG pathways in red edges are present in the EB-247.

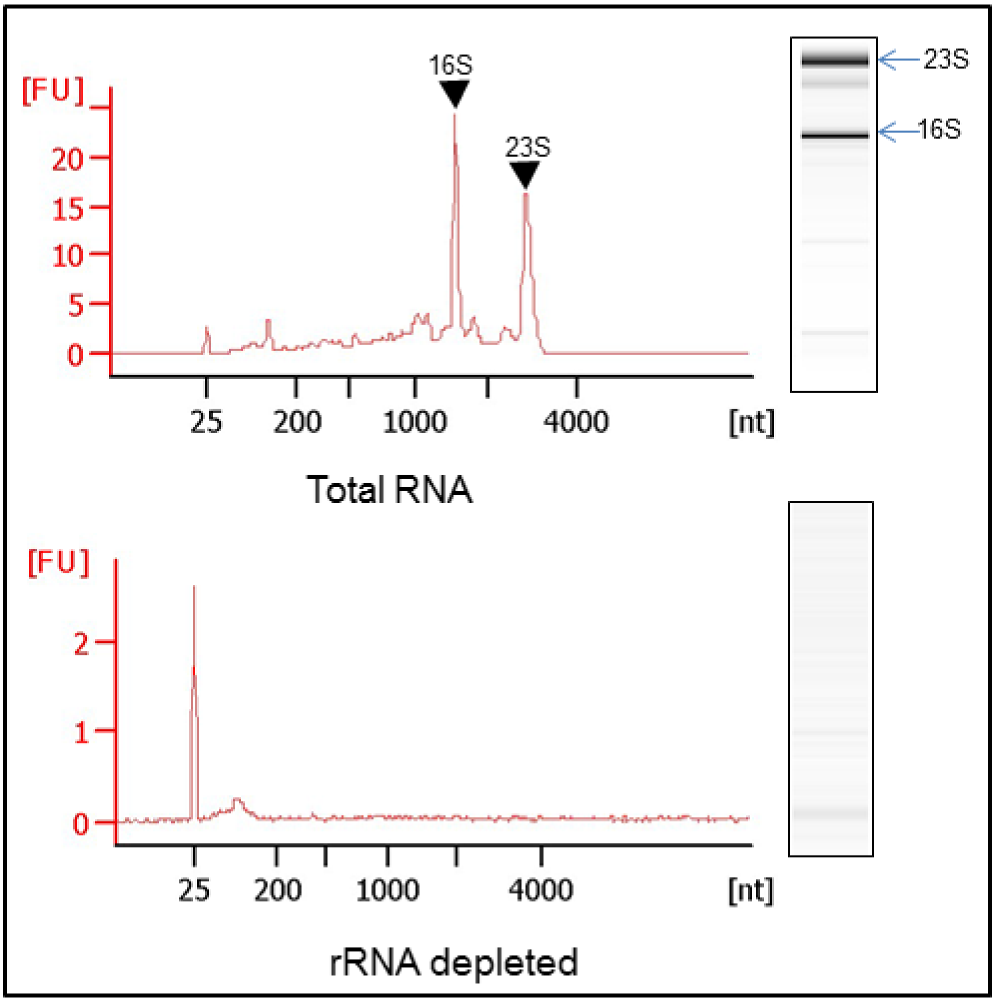

Figure S4

RNA quality analysis before preparation of sequencing library. Analysis of total RNA (upper panel) and enriched mRNA (lower panel) using BioAnalyzer 2100 (Agilent) enumeration.

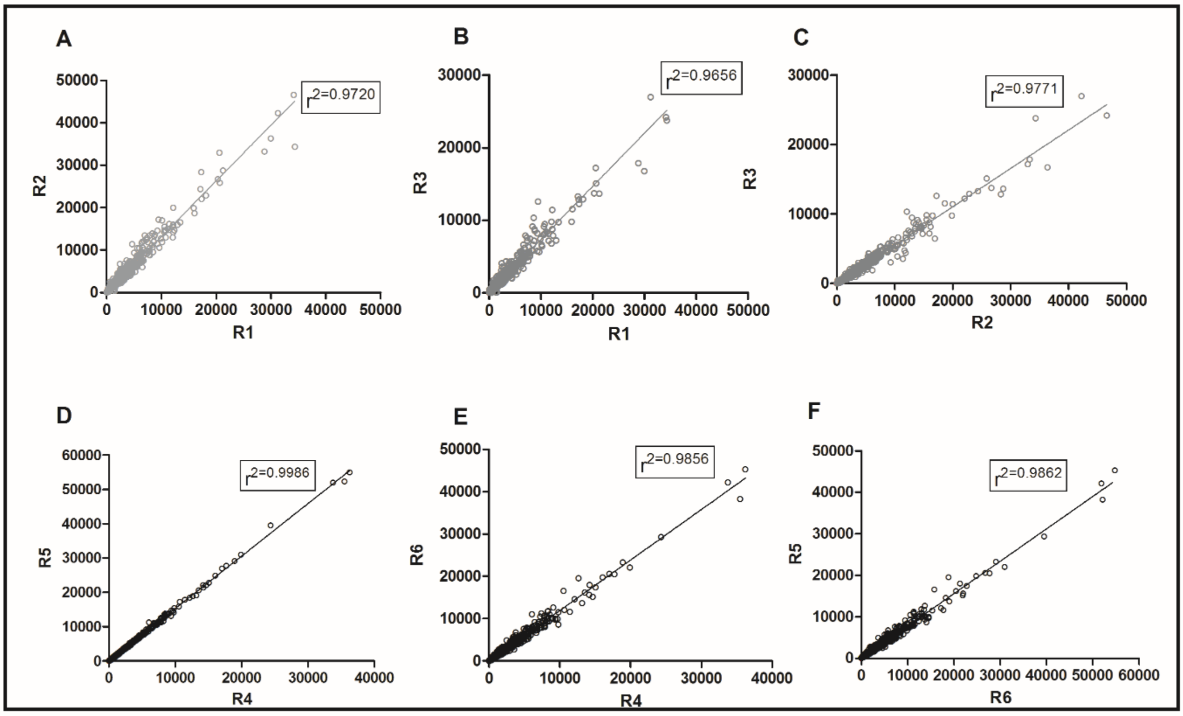

Figure S5

Summary of the sequence read data obtained from MiSeq. Correlation between the normalized reads of individual genes obtained from the sequencing of all three repeat experiments. Samples from LB are named as R1, R2 & R3 and samples from serum are named as R4, R5 & R6. The linear regression value was obtained by using the approach ‘Goodness of fit’ of GraphPad Prism4.

Table S1. Virulence factors identified in E247. *In silico* analysis of E247 genome using BLASTx (>70% query coverage, >70% protein identity and e-value <0.01) identified 72 genes (Chen et al., 2005).

Sr. No.	sseqid	Protein Idenity	e-value	Query coverage	Gene	Product
1	VFG1582	82.99	0	99.77	orf48	hypothetical protein [Escherichia coli 536]
2	VFG1583	90.56	0	100.00	orf49	hypothetical protein [Escherichia coli 536]
3	VFG1584	82.95	1E-170	97.13	orf50	hypothetical protein [Escherichia coli 536]
4	VFG0490	76.42	1E-166	99.69	orf319	putative inner membrane protein [Salmonella enterica (serovar typhimurium) LT2]
5	VFG0491	70.25	1E-109	100.00	orf242	putative regulatory proteins, merR family [Salmonella enterica (serovar typhimurium) LT2]
6	VFG0332	77.6	5E-159	100.00	rfaD	ADPL glycero D-mannoheptose 6-epimerase [Haemophilus influenzae Rd] (VF0044)
7	VFG0583	75.27	1E-37	100.00	yjcB	putative inner membrane protein [Salmonella enterica (serovar typhimurium) LT2]
8	VFG0584	73.17	0	97.19	yjcC	putative diguanylate cyclase/phosphodiesterase [Salmonella enterica (serovar typhimurium) LT2]
9	VFG0585	93.46	4E-51	100.00	soxS	transcriptional activator of superoxide response regulon (AraC/XylS family) [Salmonella enterica (serovar typhimurium) LT2]
10	VFG0586	92.11	2E-86	100.00	soxR	redoxsensing transcriptional activator SoxR, contains ironsulfur center for redoxsensing (MerR family) [Salmonella enterica (serovar typhimurium) LT2]
11	VFG1855	75.19	0	95.27	htpB	Hsp60, 60K heat shock protein HtpB [Legionella pneumophila Philadelphia 1] (VF0159)
12	VFG1536	72.42	0	98.23	intB	bacteriophage P4 integrase [Escherichia coli 536]
13	VFG1050	89.6	0	100.00	orf34	unknown [Shigella flexneri (serotype 2a) YSH6000]
14	VFG0446	74.67	0	97.13	fimD	outer membrane usher protein [Salmonella enterica (serovar typhimurium) LT2] (VF0102)
15	VFG0449	77.62	2E-99	100.00	fimZ	fimbrial protein Z, putative transcriptional regulator (LuxR/UhpA family) [Salmonella enterica (serovar typhimurium) LT2] (VF0102)
16	VFG0525	80.36	1E-148	90.16	sitA	Salmonella iron transporter: fur regulated [Salmonella enterica (serovar typhimurium) LT2]
17	VFG0526	80.99	3E-136	96.34	sitB	Salmonella iron transporter: fur regulated [Salmonella enterica (serovar typhimurium) LT2]
18	VFG0527	86.81	2E-127	100. 00	sitC	Salmonella iron transporter: fur regulated [Salmonella enterica (serovar typhimurium) LT2]
19	VFG0528	72.06	4E-116	96.45	sitD	Salmonella iron transporter: fur regulated [Salmonella enterica (serovar typhimurium) LT2]
20	VFG0923	80.9	0	101.00	fepA	Ferrienterobactin receptor precursor [Escherichia coli CFT073] (VF0228)
21	VFG0930	77.56	0	99.61	entF	Enterobactin synthetase component F [Escherichia coli CFT073] (VF0228)

22	VFG0925	87.06	8E-137	94.10	fepC	Ferric enterobactin transport ATPbinding protein fepC [Escherichia coli CFT073] (VF0228)
23	VFG0928	81.52	3E-127	100.00	fepG	Ferric enterobactin transport system permease protein fepG [Escherichia coli CFT073] (VF0228)
24	VFG0926	87.76	5E-113	86.98	fepD	Ferric enterobactin transport system permease protein fepD [Escherichia coli CFT073] (VF0228)
25	VFG0924	81.76	1E-164	100.00	fepB	Ferrienterobactinbinding periplasmic protein precursor [Escherichia coli CFT073] (VF0228)
26	VFG0931	78.93	0	99.75	entC	Isochorismate synthase entC [Escherichia coli CFT073] (VF0228)
27	VFG0932	81.05	0	99.44	entE	Enterobactin synthetase component E [Escherichia coli CFT073] (VF0228)
28	VFG0933	84.91	2E-163	100.00	entB	Isochorismatase [Escherichia coli CFT073] (VF0228)
29	VFG0934	87.9	7E-123	100.00	entA	2,3dihydro2,3dihydroxybenzoate dehydrogenase [Escherichia coli CFT073] (VF0228)
30	VFG0478	97.95	6E-89	97.33	fur	transcriptional repressor of ironresponsive genes (Fur family) (ferric uptake regulator) [Salmonella enterica (serovar typhimurium) LT2] (VF0113)
31	VFG1702	73.71	1E-99	96.67	c3565	Putative response regulator [Escherichia coli CFT073]
32	VFG1443	86.89	3E-177	100.00	ompA	outer membrane protein A [Escherichia coli] (VF0236)
33	VFG0572	92	5E-123	99.56	slsA	putative inner membrane protein [Salmonella enterica (serovar typhimurium) LT2]
34	VFG0462	93.94	2E-164	95.31	csgG	putative transcriptional regulator in curly assembly/transport, 2nd curli operon [Salmonella enterica (serovar typhimurium) LT2] (VF0103)
35	VFG0461	84.06	2E-57	100.00	csgF	curli production assembly/transport component, 2nd curli operon [Salmonella enterica (serovar typhimurium) LT2] (VF0103)
36	VFG0460	77.86	6E-55	100.00	csgE	curli production assembly/transport component, 2nd curli operon [Salmonella enterica (serovar typhimurium) LT2] (VF0103)
37	VFG0457	74.83	2E-65	100.00	csgB	minor curlin subunit precursor, nucleator for assembly of adhesive surface organelles [Salmonella enterica (serovar typhimurium) LT2] (VF0103)
38	VFG2351	81.75	1E-68	100.00	flgB	flagellar basalbody rod protein FlgB [Yersinia enterocolitica 8081] (VF0394)
39	VFG2350	79.85	2E-65	100.00	flgC	flagellar basalbody rod protein FlgC [Yersinia enterocolitica 8081] (VF0394)
40	VFG2349	70.26	6E-71	100.00	flgD	basalbody rod modification protein FlgD [Yersinia enterocolitica 8081] (VF0394)
41	VFG2513	71.54	4E-110	99.24	flgG	flagellar basal body rod protein FlgG [Burkholderia pseudomallei K96243] (VF0430)
42	VFG2346	85.25	8E-113	100.00	flgG	flagellar basalbody rod protein FlgG [Yersinia enterocolitica 8081] (VF0394)
43	VFG2345	80.63	4E-103	97.80	flgH	flagellar Lring protein precursor [Yersinia enterocolitica 8081] (VF0394)
44	VFG2344	78.61	6E-150	96.65	flgI	flagellar Pring protein precursor [Yersinia enterocolitica 8081] (VF0394)
45	VFG0474	82.1	0	91.79	phoQ	sensory kinase protein in twocomponent regulatory system with PhoP, ligand is Mg+ [Salmonella enterica (serovar typhimurium) LT2] (VF0111)

46	VFG0475	94.14	1E-127	99.11	phoP	response regulator in twocomponent regulatory system with PhoQ, transcribes genes expressed under low Mg+ concentration (OmpR family) [Salmonella enterica (serovar typhimurium) LT2] (VF0111)
47	VFG0916	80.7	0	100.00	chuS	Putative heme/hemoglobin transport protein [Escherichia coli CFT073] (VF0227)
48	VFG0917	84.44	0	100.00	chuA	Outer membrane heme/hemoglobin receptor [Escherichia coli CFT073] (VF0227)
49	VFG0479	96.74	0	97.87	pykF	pyruvate kinase I (formerly F), fructose stimulated [Salmonella enterica (serovar typhimurium) LT2]
50	VFG2070	74.16	0	95.58	PA0084	hypothetical protein [Pseudomonas aeruginosa PAO1] (VF0334)
51	VFG2069	73.17	2E-73	95.35	PA0083	hypothetical protein [Pseudomonas aeruginosa PAO1] (VF0334)
52	VFG2356	85.07	0	99.71	flhA	flagellar biosynthesis protein FlhA [Yersinia enterocolitica 8081] (VF0394)
53	VFG2525	75.4	4E-46	88.73	cheY	chemotaxis protein CheY [Burkholderia pseudomallei K96243] (VF0430)
54	VFG2526	70.82	6E-157	96.19	cheB	chemotaxisspecific methylesterase [Burkholderia pseudomallei K96243] (VF0430)
55	VFG2530	71.81	2E-52	85.14	cheW	chemotaxis protein CheW [Burkholderia pseudomallei K96243] (VF0430)
56	VFG2358	81.87	2E-99	100.00	flhC	flagellum biosynthesis transcription activator [Yersinia enterocolitica 8081] (VF0394)
57	VFG2359	78.35	2E-39	81.51	flhD	flagellar transcriptional activator [Yersinia enterocolitica 8081] (VF0394)
58	VFG2319	84.32	1E-124	98.33	fliA	RNA polymerase sigma factor for flagellar operon [Yersinia enterocolitica 8081] (VF0394)
59	VFG0669	77.5	2E-40	100.00	gtrA	Bactoprenollinked glucose translocase [Shigella flexneri (serotype 2a) 301] (VF0124)
60	VFG0670	86.84	5E-156	98.38	gtrB	bactoprenol glucosyl transferase [Shigella flexneri (serotype 2a) 301] (VF0124)
61	VFG2329	82.98	4E-169	99.70	fliG	flagellar motor switch protein FliG [Yersinia enterocolitica 8081] (VF0394)
62	VFG2331	84.65	0	100.00	fliI	flagellumspecific ATP synthase [Yersinia enterocolitica 8081] (VF0394)
63	VFG2335	84.08	0	99.70	fliM	flagellar motor switch protein FliM [Yersinia enterocolitica 8081] (VF0394)
64	VFG2336	76.81	2E-57	100.00	fliN	flagellar motor switch protein FliN [Yersinia enterocolitica 8081] (VF0394)
65	VFG2338	83.56	1E-118	98.68	fliP	flagellar biosynthetic protein FliP [Yersinia enterocolitica 8081] (VF0394)
66	VFG2362	72.44	0	98.68	manB	phosphomannomutase [Yersinia enterocolitica 8081] (VF0392)
67	VFG2364	73.44	3E-163	99.69	fcl	GDPfucose synthetase [Yersinia enterocolitica 8081] (VF0392)
68	VFG2365	78.17	0	99.73	gmd	GDPmannose 4,6dehydratase [Yersinia enterocolitica 8081] (VF0392)
69	VFG0524	86.25	0	99.86	fhlA	formate hydrogenlyase transcriptional activator for fdhF, hyc and hyp operons (EBP family) [Salmonella enterica (serovar typhimurium) LT2]
70	VFG0562	94.34	0	99.18	mutS	methyldirected mismatch repair, recognize exocyclic adducts of guanosine [Salmonella enterica (serovar typhimurium) LT2]

71	VFG0477	98.79	0	100.00	rpoS	sigma S (sigma 38) factor of RNA polymerase, major sigmafactor during stationary phase [Salmonella enterica (serovar typhimurium) LT2] (VF0112)
72	VFG0331	70.87	0	96.64	rfaE	ADPheptose synthase [Haemophilus influenzae Rd] (VF0044)

Table S2. Genes involved in multidrug resistance. *In silico* analysis of E247 genome using BLASTp (>70% query coverage, >70% protein identity and e-value <0.01) identified 72 genes. The BLASTp was performed against curated antibiotic resistance gene database (Gibson et al., 2015).

Region	Start	End	Protein identity	evalue	Querry coverage	Gene id	Gene name
Chromosome	29841	28660	87.56	7,00E-151	99,49	NC_002695.1	emrD
Chromosome	339887	340342	94.08	6,00E-88	98,70	CP000034.1	soxR
Chromosome	412293	411226	74.16	3,00E-179	94,43	NC_002695.1	ampC
Chromosome	714629	713769	97.21	0	99,31	AFK13827.1	RobA
Chromosome	1221904	1223004	95.1	0	100,00	gi|227029|	romA
Chromosome	1223007	1223378	94.35	9,00E-65	100,00	AFK13828.1	RamA
Chromosome	1584310	1585407	83.2	8,00E-179	99,46	NC_002695.1	MacA
Chromosome	1848424	1847084	81.43	0	91,98	NC_002695.1	PhoQ
Chromosome	2299840	2299469	93.55	1,00E-71	97,64	NC_002695.1	mrA
Chromosome	3132388	3133107	94.17	6,00E-134	100,00	NC_002695.1	baeR
Chromosome	3255052	3252479	92.07	0	98,06	NC_002695.1	gyrA
Chromosome	3688258	3689772	93.07	0	98,63	NC_002695.1	emrB
Chromosome	3995046	3992857	92.74	0	100,00	M58408	parC
Chromosome	4016603	4017991	87.55	0	94,52	NC_002695.1	tolC
Chromosome	4293620	4293249	93.55	5,00E-71	100,00	FQ312006.1	HIB
Plasmid	123775	124998	94.61	0	96,2	CP001485.1	tetA
Plasmid	123811	124947	77.04	8,00E-163	100	Y19114	tetC
Plasmid	149936	149079	100	6,00E-180	100	X13542	aac
Plasmid	152131	151382	99.2	3,00E-163	90,5	AF227505	blaOXA1
Plasmid	157318	156446	100	0	100	AY995206	blaCTXM-15
Plasmid	160140	160997	100	3,00E-180	100	FQ312006.1	bla
Plasmid	162566	161712	96.86	5,00E-179	94,09836066	gi|48783567|	antibiotic resistance kinase
Plasmid	163360	162560	99.63	5,00E-174	100	AF024602.1	StrA
Plasmid	169741	170547	99.26	3,00E-173	97,46	NC_010410	aadA1
Plasmid	169759	170547	99.62	3,00E-169	100	NC_010410	aadA1

Table S3. Primers used for validation of selective differentially regulated genes.

Gene name and locus no.	Sequence
Fw FhuC (0816)	5’TGGTAGGGCTAAAACCGCTG3’
Rw FhuC (0816)	5’GCAGAGGTCGGTTCATCCAG3’
Fw FhuD (0817)	5’AACCTTGAGCTTCTCACGCA3’
Rw FhuD (0817)	5’TCGCCATGATCTCTTCCGAC3
Fw HmuS (1825)	5‘TGGCGGTGACGGTTATTTCA3‘
Rw HmuS (1825)	5‘GCATACCGAACGTTTACGCC3‘
Fw hmuV (1822)	5’GCTCATCCCGTCATGGTAGG3’
Rw hmuV (1822)	5’AGCGTTGCGGAATAAACAGC3’
Fw hmuU (1823)	5’CGGAACGCATCATCAACACC3’
Rw hmuU (1823)	5’ATCAAACGTCCGACTTCCCC3’
Fw hmuT (1824)	5’GAGGATAAACAGCACCCGCT3’
Rw hmuT (1824)	5’CAACGCTACGCACCATGAAG3’
Fw FeoA (4029)	5’ ATTACCGGTTTTACCCGCGA 3‘
Rw FeoA (4029)	5’ ATGAACAGGATCGCCCAACG 3‘
Fw FeoB (4030)	5‘ TCACGTCCCGCATCTGAAAA 3‘
Rw FeoB (4030)	5’ CAGCGCGCTCAGGAAAATAC 3‘
Fw feoC (4031)	5’TCACGTCCCGCATCTGAAAA 3‘
Rw feoC (4031)	5’CAGCGCGCTCAGGAAAATAC3‘
Fw IutA (2842)	5‘TGCTTTCTACCTCTGCGTGG3‘
Rw IutA (2842)	5‘AGAGCGTTCAATGACCCAGG3‘
Fw hutA (1826)	5’CGTTGGCAAGCATCAGATCG3’
Rw hutA (1826)	5’ATTGGCCGCTTCTACACCAA3’
Fw iucA (2840)	5’GTGAAATGGTTGAGCTGGGC3’
Rw iucA (2840)	5’AGCGGCAGCTTGATGTCATA3’
Fw iucB (2839)	5’GGCGAGCGTTTCACTCAATG3’
Rw iucB (2839)	5’AGGTAGTTTTCCTGCTCGGC3’
Fw iucD (2841)	5’GCGCAAAAAGTTCTACCGCT3’
Rw iucD (2841)	5’AGGTTGGTGAGGTTGTCTGC3’
Fw iucA* (2840) 5’	5‘AAGATCTCTACGCCACGCTG3‘

Rw iucA* (2840) 5’	5'GCCGCTCATCAGACACTGTA3‘
Fw 1116 (Ferrichrome receptor)	5’GGTCGTTCGCGGGTATAACA3’
Rw 1116(Ferrichrome receptor)	5GTGGTTGGCGTAGAGGGAAA’3’
Fw 2633(Ferrichrome receptor)	5’TAAGCCGCCAAATTCCACCT3’
Rw 2633(Ferrichrome receptor)	5’TTCGCAAATCCACGAAAGCG3’
Fw 3702(Ferrichrome receptor)	5’TGTACGGCATTCTCGATCCG3’
Rw 3702(Ferrichrome receptor)	5’AGAGGTGGCAGAAATCGAGC3’
Fw 1699(Ferrichrome receptor)	5’CCACGTAAGAGCCGACGTAA3’
Rw 1699(Ferrichrome receptor)	5’TTCAGCCGCTATCAGGTCAC3’
Fw efeU (1623)	5’TGCAGAAGGGGAATCACCAC3’
Rw efeU (1623)	5’AAACCGATTCCAGACCCTCG3’
Fw efeO (1624)	5’TACGTCACCAAAGAGACGGC3’
Rw efeO (1624)	5’TTCGATATCGCCCGCTTTCA3’
Fw efeB (1625)	5’CATTACCGTGTCGGTAGGGG3’
Rw efeB (1625)	5’GAGGGAGTCATTCGGGAAGC3’
Fw FepA (1172)	5‘GCCCTGCTCTGGGATAACAA3‘
Rw FepA (1172)	5‘AGCTGTGATCGCCAATCGTA3‘
Fw fepA* (2987)	5’GTCCTCCTTCGGCTGGAAAA3‘
Rw fepA *(2987)	5’AACTGACCGCTCTTCTGCTC3‘
Fw Fes (1173)	5’TTACCACCGGGGATGAAAGC3’
Rw Fes (1173)	5’GGGCCAGACAGGCATACTTT3’
Fw entF (1175)	5’GGAGTTCTGGTGGCCGTTTA3’
Rw entF (1175)	5’CACGAAGTGGGTGGTGGTTA3’
Fw fepC (1176)	5’AGCAGCATAATGGCGGTCTC3’
Rw fepC (1176)	5’CTGGCTCAGCAAAGCGTAGA3’
Fw entS (1179)	5’TCGCTACGAGCGTAAGAAGC3’
Rw entS (1179)	5’AGACACAGCCCGATAAAGCC3’
Fw fepB (1180)	5’TTCTGTCGTCCACAGGTTCG3’
Rw fepB (1180)	5’CGCGTTCGATAAACAGCTCG3’

Fw entC (1182)	5’CGATTCTCTGAACCTGCCGT3’
Rw entC (1182)	5’CTACCATGACGGGATGAGCG3’
Fw entE (1183)	5’CCTTTGTGGATGAACACCGC3’
Rw entE (1183)	5’GTCGGGTTCGGGATGAAGTT3’
Fw entB (1184)	5’AGATGCTGAAAGAGACCGGC3’
Rw entB (1184)	5’TCGGCGATAAAGAACGGCTT3’
Fw entA (1185)	5’GACCTTTGCGGTTAACGTCG3’
Rw entA (1185)	5’GAGGCCACGGTCACTATCG3’
Fw entH (1186)	5’GCTCAATGGCCGGTTTTCTG3’
Rw entH (1186)	5’ATGTAGCGGCTGACACACTC3’
Fw tonB (1826)	5’AGCTTGCTGGTGACGGTATC3‘
Rw tonB (1826)	5’CCTCGACGTGGCCTATAACC3‘
Fw PgaA (3652)	5‘CGCATCTTCAGACGCTTTCG3‘
Rw PgaA (3652)	5‘TATCCGTTAGCGCGTCAGTC3
Fw PgaB (3654)	5‘GTGAGCCTGATCCTCATCGG3‘
Rw PgaB (3654)	5‘TAGGCCAGCACCAGAAACTG3‘
Fw PgaC (3655)	5‘CGCGGAGGTATTTCTGGTGA3‘
Rw PgaC (3655)	5‘TGATGTACAGCAGCACCGTT3‘
Fw PgaD (3656)	5‘TGGTATCAGTACAACCGCCG3‘
Rw PgaD (3656)	5‘AGCTGCGGTGAGACGTTAAA3‘
