# Supplementary material for: Enterobacter bugandensis: a novel enterobacterial species associated with severe clinical infection
Source: Sci Rep. 2018 Mar 29;8:5392. doi: 10.1038/s41598-018-23069-z (PMC5876403; doi:10.1038/s41598-018-23069-z)
Supplement: Supplementary file 4 — File S3 [file 41598_2018_23069_MOESM4_ESM.pdf]

A	Iron uptake and metabolism	
**Locus**	**Product**	**Log2FC**
Eb1183	2,3-dihydroxybenzoate-AMP ligase [enterobactin] siderophore	5,808197028
Eb1826	TonB-dependent hemin , ferrichrome receptor	5,459307208
Eb1825	Hemin transport protein HmuS	4,659399824
Eb1184	Apo-aryl carrier domain of EntB	4,634578251
Eb1182	Isochorismate synthase [enterobactin] siderophore	4,560528217
Eb2842	Aerobactin siderophore receptor IutA	4,344129795
Eb2841	aerobactin biosynthesis protein IucD	4,337210041
Eb1185	2,3-dihydro-2,3-dihydroxybenzoate dehydrogenase [enterobactin] siderophore	4,321353888
Eb1824	Periplasmic hemin-binding protein	4,177242183
Eb2987	TonB-dependent receptor; Outer membrane receptor for ferrienterochelin and colicins	4,130037753
Eb1175	Enterobactin synthetase component F, serine activating enzyme	4,037156125
Eb1823	Hemin ABC transporter, permease protein	4,023203371
Eb1822	ABC-type hemin transport system, ATPase component	3,921647724
Eb1172	TonB-dependent receptor for ferric enterobactin and colicins B, D	3,78686352
Eb2839	aerobactin biosynthesis protein IucB	3,600420746
Eb2840	aerobactin biosynthesis protein IucA	3,49201308
Eb2838	aerobactin biosynthesis protein IucA	3,182568779
Eb1186	Proofreading thioesterase in enterobactin biosynthesis EntH	3,056414834
Eb1176	Ferric enterobactin transport ATP-binding protein FepC	2,702099354
Eb1173	Enterobactin esterase	2,453105045
Eb2633	Ferrichrome-iron receptor	2,322245722
Eb1116	Ferrichrome-iron receptor	2,283464684
Eb0815	Ferric hydroxamate outer membrane receptor FhuA	2,280165591
Eb3698	Putative iron compound-binding protein of ABC transporter family	2,255098104
Eb1624	Ferrous iron transport periplasmic protein EfeO	2,23756998
Eb1179	Enterobactin exporter EntS	2,230867854
Eb1625	Ferrous iron transport peroxidase EfeB	2,141293311
Eb0816	ATP-binding protein FhuC	2,107867063
Eb4030	Ferrous iron transport protein B	1,895461005
Eb1623	Ferrous iron transport permease EfeU	1,883415459
Eb0817	periplasmic substrate binding protein FhuD	1,752679086
Eb1180	Ferric enterobactin-binding periplasmic protein FepB	1,750714241
Eb1699	Putative OMR family iron-siderophore receptor precursor	1,600669829
Eb0637	Ferric reductase	1,565302525
Eb4029	Ferrous iron transport protein A	1,551422797
Eb3998	Acetylornithine aminotransferase	1,290932914
Eb4031	Ferrous iron-sensisng transcriptional regulator FeoC	1,143614606
Eb3333	Uncharacterized iron-regulated membrane protein; Iron-uptake factor PiuB	1,005091363
Eb3977	Bacterioferritin-associated ferredoxin	0,993288165
Eb3702	Ferrichrome-iron receptor	0,988869968
Eb2752	Ferritin-like protein 2	0,979432959
Eb2244	Putative hemin- binding lipoprotein	-1,231175243
Eb2243	Putative metaloprotease associated with hemin utilization	-1,567152362

B	Regulatory functions	
**Locus**	**Product**	**Log2FC**
Eb0490	transcriptional regulator	1,189419912
Eb0499	Aspartate carbamoyltransferase regulatory chain	1,154497366
Eb2299	Transcriptional regulator, ArsR family	-0,878328895
Eb4318	Putative regulator protein	-0,909922909
Eb0372	Transcriptional regulator, TetR family	-0,938771267
Eb4026	Two-component system response regulator OmpR	-0,991088767
Eb4296	Nitrogen regulation protein	-1,158897336
Eb1667	Negative regulator of flagellin synthesis FlgM	-1,181309459
Eb3764	LsrR, transcriptional repressor of lsr operon	-1,204333542
Eb0156	Putative regulatory protein	-1,209130149
Eb1986	LysR family transcriptional regulator YnfL	-1,22452492
Eb0142	Mannitol operon repressor	-1,372519375
Eb1980	Putrescine utilization regulator	-1,409985616
Eb0063	Transcriptional regulatory protein UhpA	-1,47507443
Eb0337	Response regulator protein Z5684	-1,57823114
Eb2214	Chemotaxis regulator	-1,581440915
Eb2298	Translation elongation factor G	-1,735773763
Eb2242	Transcriptional regulators	-1,742225373
Eb2027	Transcriptional activator feaR	-1,767123661
Eb0914	Lactate-responsive regulator LldR in Firmicutes, GntR family	-2,410101796

C Chaperons

Locus	Product	Log2FC
Eb0187	Cold shock protein CspA	0,932294385
Eb0015	16 kDa heat shock protein A	0,759228942
Eb2671	Cold shock protein CspC	0,650173558

D Others

Locus	Product	Log2FC
Eb1827	putative cytoplasmic protein	2,853498958
Eb3687	Protein ygiW precursor (cellular response to cadmium)	1,599498634
Eb1293	cyd operon protein YbgT	1,364783159
Eb0550	Colicin-E2 immunity protein (ImmE2) (Microcin-E2 immunity protein)	1,265411149
Eb2612	Uncharacterized protein ImpB	1,06836953
Eb2380	Gns protein	1,038005134
Eb2517	Outer membrane protein W precursor	0,973367726
Eb2611	Uncharacterized protein ImpC	0,896747663
Eb1232	Iojap protein	0,827957503
Eb1272	Putative exported protein	0,743647744
Eb0457	Uncharacterized protein YtfM precursor	-0,790667532
Eb0178	Uncharacterized lipoprotein YsaB precursor	-0,848207212
Eb2685	protein of unknown function DUF1480	-0,903922352
Eb2412	Putative methyl-accepting chemotaxis protein	-0,995078767
Eb1160	RNA polymerase sigma-70 factor	-1,132302679
Eb2780	Flagellar biosynthesis protein FliC	-1,296001857
Eb2735	Sigma-fimbriae usher protein	-1,335540041
Eb4115	Uncharacterized protein YhjG	-1,33984084
Eb3282	Inner membrane protein YphA	-1,360725661
Eb1440	probable lipoprotein	-1,418559347
Eb2108	Protein Implicated in DNA repair function with RecA and MutS	-1,419160135
Eb2642	Stage V sporulation protein involved in spore cortex synthesis	-1,424719874
Eb1858	Iron-sulfur cluster assembly protein SufD	-1,44126834
Eb2354	Gifsy-2 prophage protein	-1,53637066
Eb2634	UPF0410 protein YmgE	-1,578420621
Eb4000	Cell filamentation protein fic	-1,580550209
Eb1627	Nicotinamidase family protein YcaC	-1,583485922
Eb2854	Cobalt-zinc-cadmium resistance protein CzcA	-1,606475624
Eb1747	UPF0229 protein YeaH	-1,631113334
Eb2737	Sigma-fimbriae uncharacterized paralogous subunit	-1,687831134
Eb1557	UPF0319 protein YccT precursor	-1,715695382
Eb0778	Putative exported protein	-1,726169117
Eb2327	Putative virulence effector protein	-1,764166361
Eb3913	Putative sulfite oxidase subunit YedY	-1,766525947
Eb2738	Sigma-fimbriae uncharacterized paralogous subunit	-1,795478554
Eb3897	probable ribonuclease inhibitor YPO3690	-1,820808507
Eb3365	Large repetitive protein	-1,859393842
Eb1187	Carbon starvation protein A	-1,953736408
Eb3280	Stationary phase inducible protein CsiE	-1,972461727
Eb4100	Universal stress protein B	-2,042771133
Eb2806	putative cytoplasmic protein	-2,182050427
Eb1626	Phosphate starvation-inducible protein PhoH	-2,227294784
Eb0426	Putative exported protein	-2,246755931
Eb2687	Virulence factor VirK	-2,526652784
Eb1471	Virulence factor VirK	-2,734345484

E	Transport and Binding	
Locus	Product	Log2FC
Eb3788	Serine transporter	4,574458571
Eb3047	Tricarboxylate transport membrane protein TctA	3,538502297
Eb3045	Tricarboxylate transport protein TctC	3,398641522
Eb3046	Tricarboxylate transport protein TctB	2,53710608
Eb1435	Arginine ABC transporter, periplasmic arginine-binding protein ArtJ	2,302156445
Eb0431	Ascorbate-specific PTS system, EIIB component	2,018540605
Eb0258	Maltose operon periplasmic protein MalM	2,016905773
Eb2522	Ferric siderophore transport system, periplasmic binding protein TonB	1,95398873
Eb0430	Ascorbate-specific PTS system, EIIC component	1,925437222
Eb0432	Ascorbate-specific PTS system, EIIA component	1,888175245
Eb0257	Maltoporin (maltose/maltodextrin high-affinity receptor,	1,886597593
Eb0256	Maltose/maltodextrin transport ATP-binding protein MalK	1,786911666
Eb1950	Aminobenzoyl-glutamate transport protein	1,741075986
Eb3675	Biopolymer transport protein ExbD/TolR	1,569756998
Eb2088	Outer membrane transporter, type I secration	1,535561708
Eb3448	ABC transporter (iron.B12.siderophore.hemin),	1,492287449
Eb2140	Urea ABC transporter, urea binding protein	1,490630191
Eb0255	Maltose/maltodextrin ABC transporter,	1,471831955
Eb3676	MotA/TolQ/ExbB proton channel family protein	1,447597183
Eb3450	ABC transporter (iron.B12.siderophore.hemin), ATP-binding component	1,246268148
Eb2857	Shikimate transporter	1,147446494
Eb1718	ABC transporter, periplasmic spermidine putrescine-binding protein PotD	1,105381424
Eb2866	Putrescine importer	1,024018717
Eb2753	Putative Dcu family, anaerobic C4-dicarboxylate transporter	0,940525948
Eb0091	Sodium/glutamate symport protein	0,936345803
Eb1992	Putative ABC transporter periplasmic binding protein	0,875889372
Eb0184	ABC transporter ATP-binding protein	0,681240752
Eb0705	Glutathione-regulated potassium-efflux system protein KefC	-0,895590439
Eb2761	Branched-chain amino acid ABC transporter, amino acid-binding protein	-1,061207437
Eb0444	D-serine/D-alanine/glycine transporter	-1,066436885
Eb3662	Oligopeptide ABC transporter, periplasmic oligopeptide-binding protein OppA	-1,09479132
Eb1304	Zinc transporter ZitB	-1,095240492
Eb3394	L-proline glycine betaine ABC transport system permease protein ProV	-1,13897285
Eb3763	Autoinducer 2 (AI-2) ABC transport system	-1,198369637
Eb0461	Putative sugar ABC transport system,	-1,255032773
Eb0770	Aromatic amino acid transport protein AroP	-1,294195639
Eb1240	Glutamate Aspartate transport system permeaseprotein GltK	-1,308795422
Eb0144	PTS system, mannitol-specific IIA/B/C component	-1,32686909
Eb1242	Glutamate Aspartate periplasmic binding protein precursor GltI	-1,349787709
Eb3931	Glutamate Aspartate transport system permease protein GltK	-1,442434052
Eb2336	Spermidine Putrescine ABC transporter permease component potC	-1,530550638
Eb1984	Putrescine importer	-1,539909428
Eb3930	Glutamate Aspartate transport system permease protein GltJ	-1,544286575
Eb0916	Oligopeptide transport ATP-binding protein OppF	-1,559578652
Eb1241	Glutamate Aspartate transport system permease protein GltJ	-1,563487627
Eb3929	Glutamate Aspartate periplasmic binding protein precursor GltI	-1,598515648
Eb0020	PTS system, maltose and glucose-specific IIC component	-1,626170126
Eb0341	putative periplasmic ribose-binding protein of ABC transport system	-1,803286328
Eb2338	Putrescine transport ATP-binding protein PotA	-1,916911453
Eb2339	ABC transporter, periplasmic spermidine putrescine-binding protein PotD	-2,057016861
Eb0146	Membrane fusion component of tripartite multidrug resistance system	-2,079153674
Eb3123	Long-chain fatty acid transport protein	-2,093430001
Eb2337	Spermidine Putrescine ABC transporter permease component PotB	-2,100697183
Eb3097	Lysine-arginine-ornithine-binding periplasmic protein precursor	-2,209968141
Eb0189	2-ketogluconate transporter	-2,410054203
Eb0915	Oligopeptide transport ATP-binding protein OppD	-2,410752569
Eb1270	Potassium-transporting ATPase B chain	-2,55128642
Eb1269	Potassium-transporting ATPase C chain	-2,568392153
Eb0343	Ribose ABC transport system, ATP-binding protein RbsA	-2,643883513
Eb1268	Osmosensitive K+ channel histidine kinase KdpD	-2,714423442
Eb1271	Potassium-transporting ATPase A chain	-2,834166359

F	Energy metabolism	
**Locus**	**Product**	**Log2FC**
Eb1394	Pyruvate formate-lyase	4,415193121
Eb1395	Pyruvate formate-lyase activating enzyme	3,431928235
Eb0138	L-lactate dehydrogenase	3,210115012
Eb4151	5-methyltetrahydropteroyltriglutamate-- homocysteine methyltransferase	3,139277466
Eb1034	Cysteine synthase B	2,850412807
Eb0429	Probable L-ascorbate-6-phosphate lactonase UlaG	2,729436245
Eb0140	L-lactate permease	2,514282418
Eb2139	Methionine synthase II (cobalamin-independent)	2,479321716
Eb4210	Argininosuccinate lyase	1,857419547
Eb1951	Catalyzes the cleavage of p-aminobenzoyl- glutamate to p-aminobenzoate and glutamate	1,849485983
Eb2073	5-methyltetrahydropteroyltriglutamate--homocysteine methyltransferase	1,787663008
Eb3792	Threonine dehydratase, catabolic	1,752287547
Eb0434	L-ribulose-5-phosphate 3-epimerase UlaE	1,706120126
Eb0433	3-keto-L-gulonate-6-phosphate decarboxylase UlaD	1,683627901
Eb3392	Ribonucleotide reductase of class Ib (aerobic), alpha subunit	1,680367124
Eb3829	Argininosuccinate synthase	1,649500476
Eb4211	Acetylglutamate kinase	1,57948829
Eb0236	Malate synthase	1,556090617
Eb1952	Catalyzes the cleavage of p-aminobenzoyl- glutamate to p-aminobenzoate and glutamate	1,465526494
Eb0237	Isocitrate lyase	1,437589134
Eb4212	N-acetyl-gamma-glutamyl-phosphate reductase	1,423778681
Eb0435	L-ribulose-5-phosphate 4-epimerase UlaF	1,336675048
Eb2876	Phosphoribosyl-AMP cyclohydrolase	1,311157707
Eb2869	ATP phosphoribosyltransferase	1,310551492
Eb2871	Histidinol-phosphate aminotransferase	1,272073819
Eb3513	N-acetylglutamate synthase	1,186714696
Eb2316	L-asparagine permease	1,185079203
Eb3793	Threonine catabolic operon transcriptional activator TdcA	1,179338037
Eb0702	Carbamoyl-phosphate synthase large chain	1,160367639
Eb4192	Ketol-acid reductoisomerase	1,123690465
Eb4220	Aspartokinase (EC 2.7.2.4) / Homoserine dehydrogenase	1,052394922
Eb2870	Histidinol dehydrogenase	1,042728429
Eb4050	Aspartate-semialdehyde dehydrogenase	1,03320709
Eb0500	Aspartate carbamoyltransferase	1,024499499
Eb0361	Alkylphosphonate utilization operon protein PhnA	0,970255644
Eb2872	Histidinol-phosphatase	0,911670317
Eb2873	Imidazole glycerol phosphate synthase amidotransferase subunit	0,806211362
Eb1943	NAD(P) transhydrogenase alpha subunit	0,725065707
Eb0786	Glucose dehydrogenase, PQQ-dependent	-0,859832042
Eb0274	Gamma-aminobutyrate:alpha-ketoglutarate aminotransferase	-0,893884235
Eb0114	UDP-glucose:(heptosyl) LPS alpha1,3-glucosyltransferase WaaG	-0,903512113
Eb0825	Deoxyguanosinetriphosphate triphosphohydrolase	-0,921162144
Eb4049	1,4-alpha-glucan (glycogen) branching enzyme	-0,965468443
Eb0294	Gluconate utilization system Gnt-I transcriptional repressor	-0,965515871
Eb0738	rRNA small subunit methyltransferase H	-0,975601113
Eb2045	Glutathione peroxidase family protein	-0,984379515
Eb1464	Pyruvate oxidase	-1,020791275
Eb2347	Pyruvate-flavodoxin oxidoreductase	-1,024087408
Eb3999	Para-aminobenzoate synthase, amidotransferase component	-1,11474476
Eb4025	Osmolarity sensory histidine kinase EnvZ	-1,160036856
Eb2292	Respiratory nitrate reductase beta chain	-1,225948078
Eb3200	YpfJ protein, zinc metalloprotease superfamily	-1,240580278
Eb1442	N-acetylmuramoyl-L-alanine amidase	-1,244266757
Eb2125	putative transferase clustered with tellurite resistance proteins TehA/TehB	-1,300446914
Eb1985	Permeases of the major facilitator superfamily	-1,315274251
Eb0021	Maltose-6'-phosphate glucosidase	-1,342444807
Eb0320	Glutathione S-transferase	-1,345847597
Eb4295	Dihydroxy-acid dehydratase	-1,346286843
Eb0329	Acetyl-coenzyme A synthetase	-1,351384502
Eb2290	Respiratory nitrate reductase alpha chain	-1,353615993
Eb2807	Putative mannosyl-3-phosphoglycerate phosphatase	-1,409697354
Eb1922	L-arabinose 1-dehydrogenase	-1,421370139
Eb0143	Mannitol-1-phosphate 5-dehydrogenase	-1,468444465
Eb1384	6-phospho-beta-glucosidase	-1,480967186
Eb4253	L-rhamnose operon regulatory protein RhaS	-1,508031507
Eb0604	4-hydroxyphenylacetate 3-monooxygenase	-1,511252549
Eb1896	Putative oxidoreductase	-1,539084378
Eb2957	HTH-type transcriptional regulator mlrA	-1,542811331
Eb0151	Aldehyde dehydrogenase B	-1,549206535
Eb0339	Putative aldolase Z5687	-1,589727859
Eb3810	Oxidoreductase	-1,590921705
Eb1982	Gamma-glutamyl-GABA hydrolase	-1,596129592
Eb1981	Gamma-glutamyl-GABA hydrolase	-1,605584444

Eb1748	Serine protein kinase	-1,61389503
Eb0188	2-ketoaldonate reductase, broad specificity	-1,680516697
Eb2653	Long-chain-fatty-acid--CoA ligase	-1,725997173
Eb3898	Succinate-semialdehyde dehydrogenase [NADP+]	-1,758040363
Eb2043	Phenylacetic acid degradation operon negative regulatory protein PaaX	-1,809780504
Eb3120	Enoyl-CoA hydratase	-1,819847341
Eb3121	3-ketoacyl-CoA thiolase	-1,830429169
Eb0340	ATP synthase delta chain	-1,842702239
Eb2217	Cellulose synthase	-1,856894301
Eb0145	GTPase	-1,886761333
Eb1983	Gamma-glutamyl-putrescine synthetase	-1,912062832
Eb0807	Sugar/maltose fermentation stimulation protein homolog	-1,926819129
Eb0191	Epimerase KguE	-1,989262601
Eb4261	Alpha-galactosidase	-2,018005334
Eb4137	Acetyl-CoA acetyltransferase	-2,041496143
Eb1778	Succinylglutamate desuccinylase	-2,081377132
Eb1776	Succinylglutamic semialdehyde dehydrogenase	-2,092778776
Eb3748	Putrescine aminotransferase	-2,136221897
Eb0922	Butyryl-CoA dehydrogenase	-2,141884551
Eb1895	Phosphoethanolamine transferase EptA specific	-2,142951056
Eb0190	2-ketogluconate kinase	-2,263852689
Eb1777	Succinylarginine dihydrolase	-2,301908473
Eb1774	Succinylornithine transaminase	-2,359452986
Eb1775	Arginine N-succinyltransferase	-2,59169685
Eb2042	Phenylacetate-coenzyme A ligase PaaF	-2,622418004
Eb2040	Phenylacetic acid degradation protein PaaD	-2,771673177
Eb2041	3-ketoacyl-CoA thiolase	-2,937742236
Eb3789	2-ketobutyrate formate-lyase	-2,959512195
Eb2038	Phenylacetate degradation enoyl-CoA hydratase PaaB	-3,00610766
Eb2036	Phenylacetate-CoA oxygenase/reductase, PaaK	-3,198618263
Eb2035	Phenylacetate-CoA oxygenase, PaaJ subunit	-3,322870823
Eb2031	Aldehyde dehydrogenase , PaaZ	-3,408759536
Eb2032	Phenylacetate-CoA oxygenase, PaaG subunit	-3,513491839
Eb2034	Phenylacetate-CoA oxygenase, PaaI subunit	-3,611726619
Eb2033	Phenylacetate-CoA oxygenase, PaaH subunit	-3,64104643
Eb4083	UDP-4-amino-4-deoxy-L-arabinose--oxoglutarate aminotransferase	-3,661842546
Eb1394	Pyruvate formate-lyase	-3,726115012
Eb1395	Pyruvate formate-lyase activating enzyme	-3,782800557
Eb0138	L-lactate dehydrogenase	-3,839486103
Eb4151	5-methyltetrahydropteroyltriglutamate-- homocysteine methyltransferase	-3,896171648
Eb1034	Cysteine synthase B	-3,952857193
Eb0429	Probable L-ascorbate-6-phosphate lactonase UlaG	-4,009542739
Eb0140	L-lactate permease	-4,066228284
Eb2139	Methionine synthase II (cobalamin-independent)	-4,122913829
Eb4210	Argininosuccinate lyase	-4,179599375
Eb1951	Catalyzes the cleavage of p-aminobenzoyl- glutamate to p-aminobenzoate and glutamate	-4,23628492
Eb2073	5-methyltetrahydropteroyltriglutamate--homocysteine methyltransferase	-4,292970466

G	Membrane protein	
Locus	Product	Log2FC
Eb3787	Putative inner membrane protein	4,135133479
Eb1529	Outer membrane protein F precursor	1,498468068
Eb3652	Biofilm PGA outer membrane secretin PgaA	1,176295432
Eb1351	Predicted membrane fusion protein component of efflux pump	0,937663311
Eb4089	Integral membrane protein TerC	0,855360061
Eb1199	Probable glutathione S-transferase-related transmembrane protein	-0,898030785
Eb4113	Inner membrane protein YhjD	-1,18422342
Eb0153	Putative outer membrane protein yiaT precursor	-1,255129383
Eb0592	Putative inner membrane protein	-1,434022653
Eb1506	Putative inner membrane protein	-1,533583084
Eb0391	Outer membrane lipoprotein Blc	-1,646346704
Eb2967	Putative membrane protein	-1,778607539
Eb2853	Probable Co/Zn/Cd efflux system membrane fusion protein	-1,781457789
Eb0392	Outer membrane lipoprotein Blc	-1,86724431
